# Somatic mutations as markers of outcome after azacitidine and allogeneic stem cell transplantation in higher-risk myelodysplastic syndromes

**DOI:** 10.1038/s41375-018-0284-9

**Published:** 2018-10-05

**Authors:** Giulia Falconi, Emiliano Fabiani, Alfonso Piciocchi, Marianna Criscuolo, Luana Fianchi, Elisa L. Lindfors Rossi, Carlo Finelli, Elisa Cerqui, Tiziana Ottone, Alfredo Molteni, Matteo Parma, Stella Santarone, Anna Candoni, Simona Sica, Giuseppe Leone, Francesco Lo-Coco, Maria Teresa Voso

**Affiliations:** 1 Department of Biomedicine and Prevention, University of Rome Tor Vergata, Roma, Italy; 2 Fondazione GIMEMA, Roma, Italy; 3 Dipartimento Scienze Radiologiche Radioterapiche ed Ematologiche, Fondazione Policlinico Universitario A. Gemelli, IRCCS, Roma, Italy; 4 Department of Hematology, Ospedale Sant’Orsola Malpighi, University of Bologna, Bologna, Italy; 5 Department of Hematology, A.O. Spedali Civili, Brescia, Italy; 6 Department of Hematology, Ospedale Niguarda, Milano, Italy; 7 Department of Hematology, HSCT Adult Unit, San Gerardo Hospital, Monza, Italy; 8 Department of Hematology, Centro Trapianti Midollo Osseo, Pescara, Italy; 9 Division of Hematology and BMT, Department of Experimental and Clinical Medical Sciences, Azienda Ospedaliero-Universitaria di Udine, Udine, Italy; 10 Fondazione Santa Lucia, Laboratorio di Neuro-Oncoematologia, Roma, Italy

**Keywords:** Medical research, Risk factors

Somatic mutations have been shown to play a significant prognostic role in myelodysplastic syndromes (MDS). Actually, detection of a TP53, EZH2, RUNX1, ASXL1, or ETV6 mutation predicts rapid disease progression and may direct treatment choices in all MDS subgroups, also in the context of allogeneic stem cell transplantation (HSCT) [1–3], which to date remains the only curative option for higher-risk MDS (HR-MDS). We recently reported the results of the phase II multicentre BMT-AZA trial, which was designed to assess the feasibility of HSCT in HR-MDS and low-blast count acute myeloid leukemia (LBC-AML) after a short bridge with azacitidine (AZA) [4]. In this trial, hematopoietic cell transplantation-comorbidity index at the time of HSCT and response to AZA were independent predictors of overall survival (OS), underlining the importance of disease-debulking before HSCT.

We were interested in the identification of biologic predictors of response to AZA and survival, which could be used to address upfront treatment in MDS. To this purpose, we studied the prognostic role of somatic mutations and of changes in mutation burden in 65 patients (53 de novo HR-MDS and 12 LBC-AML, 21 females and 44 males, median age: 59 years, range 21–66), enrolled in the BMT-AZA trial (EudraCT number 2010-019673-15) [4]. Patients were included in the translational study according to availability of paired samples collected before treatment start and after four cycles of AZA. Main patient characteristics are shown in supplementary Table 1. All patients were treated with the standard AZA regimen (75 mg/sqm/day sc for seven days every 28 days), for a median of four cycles (range 1–11), followed by HSCT in 44 patients. Distribution of patients according to treatment and response is shown in Supplementary Figure 1 and supplementary text. Patients gave informed consent according to institutional guidelines and the declaration of Helsinki. The study had been approved by the institutional ethical committees of participating centers and of University of Rome Tor Vergata.

Ultra-deep next generation sequencing (NGS) was performed on 65 DNA samples obtained before AZA treatment start, using the commercial Myeloid Solution produced by SOPHiA GENETICS (SOPHiA GENETICS, Saint-Sulpice, Switzerland) on a HiSeq® sequencing platform (Illumina, San Diego, California). Thirty genes known to be involved in MDS and AML pathogenesis were studied (10 full genes and 20 hot-spot regions). Details on the NGS pipeline are reported as supplementary text. NGS mutation burden in cases with variant allele frequency (VAF) > 5% was validated by pyrosequencing assays (detailed in Supplementary text and Supplementary Figure 2A).

At the time of protocol enrolment, we identified at least one mutation at a VAF greater than 1%, in 62 out of 65 patients (95.4%) (Fig. 1a). The median number of mutated genes was three per patient (range, 0–6). The most commonly mutated genes were: *ASXL1* (37%), *RUNX1* (29%), *SETBP*1 (25%), *DNMT3A* (21%), *TET2* (21%), *SRSF2* (17%) and *TP53* (17%). Thirty-one of 62 patients had more than one mutation in the same gene. There were no differences in the median number of mutated genes between HR-MDS and LBC-AML patients (data not shown). A comprehensive list of all mutations identified, their localization and VAF% are reported in supplementary Table 2, while significant associations between different mutations and clinical characteristics of patients are reported in Fig. 1b and Supplementary text.

**Fig. 1:**
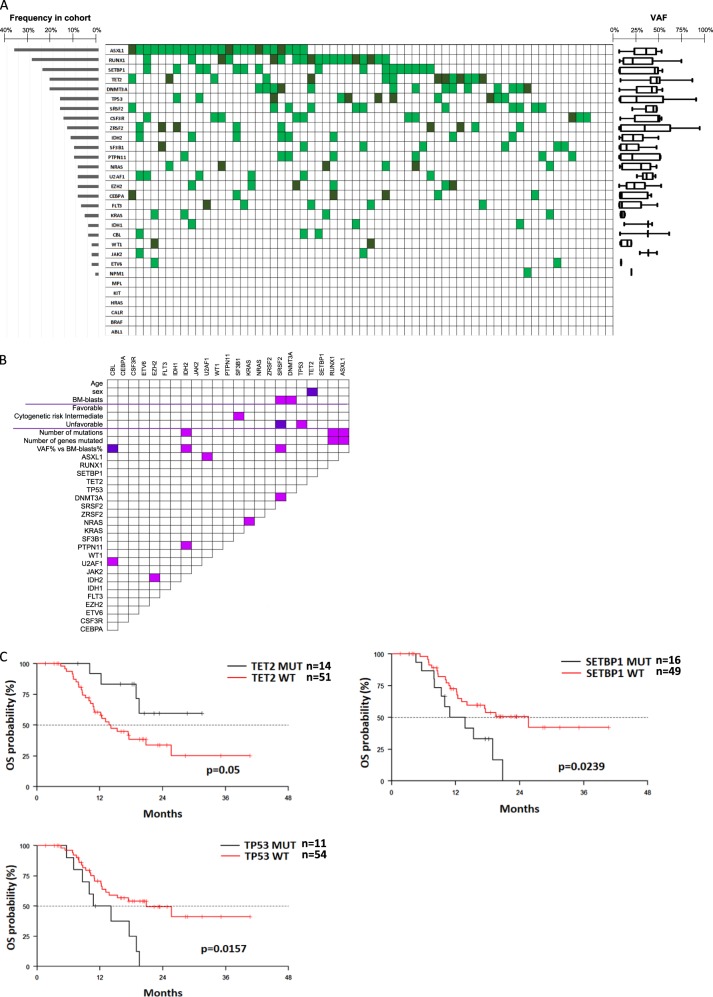
**a** Distribution, frequency and variant allele frequency (VAF) of mutations in the study cohort. Each column represents a single patient. Light- and dark- green boxes indicate the presence of 1 or ≥2 mutations in the same gene, whereas empty boxes indicate wild-type genes. Median VAF and standard deviation for each mutation are shown on the right. **b** Associations between mutations and patient characteristics. Violet and pink boxes indicate a significant negative or positive association between variables, respectively (*p* < 0.05). R Pearson test, Fisher exact test and Wilcoxon were used, according to the variables analyzed. **c** Association between OS and TET2, SETBP1 and TP53 mutations

In our cohort of 65 patients, overall response to AZA treatment was 46% (including complete remission (CR), partial remission (PR) or haematological improvement (HI) in MDS and CR/PR in LBC-AML), while patients with stable disease (SD) and progressive disease (PD) were considered unresponsive. Univariate analyses of the impact of mutational status on response according to VAF are summarized in Supplementary Figure 3. Mutations of DNMT3A localized in the functional methyl-transferase domain played a significant role for AZA response: ten of 11 patients with these mutations were unresponsive to AZA and only one achieved HI (*p* = 0.0281). In particular, all seven patient carriers of the specific DNMT3A-R882 mutation were resistant to AZA (*p* = 0.0126). Similarly, the genomic localization of SETBP1 mutations was predictive of response: all seven patients mutated in the SKI homologous region (amino acids 868–872) were resistant to AZA treatment (*p* = 0.0126). Finally, we observed that SRSF2 mutations were more frequent in patients with PD after AZA (11.3% vs 41.7%, *p* = 0.035). All other mutations, including those affecting TP53, were not predictive of AZA response. No differences in the mutational profile was observed comparing patients with MDS in SD vs PD (data not shown).

We used specific pyrosequencing assays (supplementary table 3) to quantify changes in the mutational burden of selected genes after four AZA cycles. The allelic frequency of most mutations did not change upon AZA treatment (supplementary Figure 4A). Conversely, we observed a statistically significant decrease in TP53 mutational burden (median VAF: 29.5% vs 10.5%, *p* = 0.0243, supplementary Figure 4B), which was independent of the depth of response (CR vs PR, vs HI, supplementary Figure 4C). Interestingly, in ID32 with two different TP53 mutations, one clone was sensitive and the other resistant to AZA, while the TP53 mutation burden remained unchanged for two different TP53 mutations in ID72, who progressed under AZA.

At a median follow-up of 20.3 months (1.6–40.6) after AZA start, median progression-free survival (PFS) was 12.2 months, while OS was 17.6 months. Similar to the reported extended cohort [4], patients who achieved CR, PR, HI, or SD had a longer OS as compared to patients with PD, confirming the important role of AZA induction before HSCT. In agreement with previous reports [5], patients with mutations in more than three genes had poorer OS and PFS (*p* = 0.069 and p = 0.036, supplementary Figure 5). Table 1 shows univariate and multivariate analysis for OS and PFS. In multivariate analysis, TP53 mutations were independent negative predictors for both OS and PFS (*p* = 0.0008 and *p* = 0.0013, respectively, Fig. 1c). This was independent of both VAF (median 31%, range 1–93%, supplementary Figure 6), and co-existence of more than one TP53 mutation or other mutations in the same patient. Moreover, mutations in SETBP1 were associated not only to AZA resistance, but also to decreased OS (*p* = 0.0241), whereas TET2 mutations were a favourable prognostic factor for OS (*p* = 0.0237) (Fig. 1c). The prognostic role of SETBP1 and TET2 mutations was independent from the VAF% (median 43 and 46%, range 1-52% and 3–88%, respectively). In patients who underwent HSCT (*n* = 44), TP53 and ZRSF2 mutations were a negative prognostic factor for OS after transplant (*p* = 0.014 and *p* = 0.002, respectively).

**Table 1:** OS and PFS according to mutational profiling

Parameter	Overall survival				Progression-free survival			
	Univariate analysis		Multivariate analysis		Univariate analysis		Multivariate analysis	
	HR (95%CI)	*p*-value	HR (95%CI)	*p*-value	HR (95%CI)	*p*-value	HR (95%CI)	*p*-value
Female vs male	0.784 (0.36–1.708)	0.541			1.131 (0.578–2.213)	0.7189		
R-IPSS	1.728 (1.011–2.955)	0.0455			1.51 (0.935–2.44)	0.0921		
AGE	1.013 (0.973–1.054)	0.5431			1.02 (0.983–1.058)	0.3003		
WBC	1.024 (0.987–1.064)	0.2069			1.027 (1.001–1.053)	0.0397	1.042 (1.012–1.073)	0.0062
KAR good vs poor	0.588 (0.266–1.301)	0.1901			0.603 (0.289–1.258)	0.1778		
KAR intermediate vs poor	0.831 (0.276–2.499)	0.7415			0.815 (0.323–2.057)	0.6644		
CR/PR/HI VS SD/PD	0.373 (0.175–0.796)	0.0108	0.344 (0.159–0.745)	0.0068	0.315 (0.158–0.628)	0.001	0.264 (0.129–0.541)	0.0003
HSCT	0.399 (0.177–0.900)	0.0267			0.473 (0.181–1.231)	0.1249		
ASXL1 WT VS MUT	0.715 (0.348–1.472)	0.3628			0.89 (0.465–1.704)	0.7254		
CEBPA WT VS MUT	4.155 (0.565–30.546)	0.1618			2.194 (0.527–9.133)	0.2802		
CSF3R WT VS MUT	1.051 (0.367–3.01)	0.9256			0.838 (0.35–2.009)	0.692		
DNMT3A WT VS MUT	0.774 (0.334–1.798)	0.5519			0.53 (0.257–1.092)	0.085		
DNMT3A-R882 WT VS MUT	0.374 (0.125–1.121)	0.0790			0.339 (0.137–0.836)	0.0188		
ETV6 WT VS MUT	0.636 (0.191–2.11)	0.4591			0.704 (0.249–1.993)	0.5084		
EZH2 WT VS MUT	2.615 (0.356–19.223)	0.3448			0.923 (0.283–3.01)	0.8943		
FLT3 WT VS MUT	1.116 (0.338–3.681)	0.8568			1.222 (0.375–3.979)	0.7398		
IDH2 WT VS MUT	0.433 (0.151–1.247)	0.1209			0.496 (0.206–1.195)	0.1179		
KRAS WT VS MUT	0.625 (0.189–2.067)	0.4411			0.862 (0.264–2.812)	0.8059		
NRAS WT VS MUT	4.155 (0.563–30.638)	0.1624			1.577 (0.482–5.154)	0.4511		
PTPN11 WT VS MUT	0.647 (0.225–1.859)	0.4185			0.682 (0.265–1.756)	0.4279		
RUNX1 WT VS MUT	0.783 (0.358–1.709)	0.5388			0.919 (0.454–1.862)	0.8148		
SETBP1 WT VS MUT	0.424 (0.201–0.893)	0.0239	0.420 (0.197–0.893)	0.0241	0.526 (0.268–1.031)	0.0612		
SETBP1 SKI DOMAIN WT VS MUT	0.548 (0.190–1.582)	0.2662			0.523 (0.217–1.258)	0.1478		
SF3B1 WT VS MUT	2.156 (0.514–9.036)	0.2934			1.781 (0.547–5.797)	0.3377		
SRSF2 WT VS MUT	0.701 (0.287–1.712)	0.4352			0.514 (0.235–1.122)	0.0948		
TET2 WT VS MUT	2.861 (1–8.188)	0.05	3.573 (1.185–10.773)	0.0237	1.793 (0.749–4.293)	0.19		
TP53 WT VS MUT	0.38 (0.173–0.833)	0.0157	0.225 (0.094–0.537)	0.0008	0.463 (0.217–0.988)	0.0463	0.255 (0.111–0.585)	0.0013
U2AF1 WT VS MUT	0.392 (0.15–1.026)	0.0563			0.628 (0.245–1.614)	0.3342		
ZRSF2 WT VS MUT	0.351 (0.138–0.893)	0.0279			0.426 (0.191–0.949)	0.0369		

Mutations present in less than four patients were excluded from the analysis

In recent years, the prognostic role of mutational profiling has been extensively studied in MDS and AML patients, often with controversial results due to heterogeneity in treatment context and patient subsets [1–3, 5–7]. Our analysis included younger, newly diagnosed HR-MDS or LBC-AML, homogeneously treated with AZA as bridge to HSCT. We found that the recurrent DNMT3A R882^MUT^, which occurred in a minor proportion of our patients (11%) and exerts a dominant-negative effect on the methyltransferase activity [8, 9], was significantly associated to resistance to AZA. The 'hypomethylator' phenotype associated to this mutation may explain the lack of response to hypomethylating treatment (HMT). In line with the data recently reported by Jongen-Lavrencic et al. in a wide population of AML and HR-MDS patients treated with chemotherapy [10], AZA was unable to clear the DNMT3A mutation burden in our patients. In addition, we observed for the first time, that SETBP1^SKI-domain-MUT^ was a predictor of AZA resistance. Accordingly, Winkelmann et al., showed that patients with myeloid neoplasms and SETBP1-hotspot mutations presented with rapidly evolving disease and inferior overall survival, as compared to patients with other SETBP1 mutations [11].

Although not predictive of AZA response, TP53 mutations were an unfavourable prognostic factor for survival. These data are in agreement with those reported by Craddock et al. who did not find any association between mutations studied before treatment start and response to AZA [7]. In keeping with our observations, several studies showed that TP53 mutations were independently associated with shorter survival and shorter time to relapse in patients undergoing HSCT, regardless of the induction or conditioning regimens used [1–3, 6]. On the contrary, Welch et al. reported that decitabine (DAC) at the extended ten-day dosing was able to reset TP53-mutations in patients with AML or MDS [12]. In this context, DAC *bridge* nullified the prognostic role of unfavourable karyotype and TP53 mutations. The different results described in patients receiving AZA versus those treated with DAC may be due to a more pronounced or specific cytotoxic action of prolonged DAC on TP53^mut^ clones, which may not be reproduced by AZA at the standard schedule.

The role of TP53 allelic burden is controversial. Sallman et al., identified the TP53^mut^ 40% cut-off as predictor of poor survival [13]. Similar to Lindsley et al.[2], the negative prognostic role of TP53^mut^ for survival in our patients was independent of VAF and of the number of concomitant mutations. In our study, although the TP53^mut^ allelic burden significantly decreased upon AZA induction, TP53 mutations never became undetectable, also in patients achieving CR. Small TP53^mut^ clones may be sufficient to drive relapse or progression after HSCT. DAC may be more appropriate than AZA in TP53-mutated patients with MDS, and addition of targeted treatments may be envisaged in the context of a personalized medicine approach to further reduce the relapse risk.

## Electronic supplementary material

Supplementary text

Supplementary Tables

Supplementary figures
