# Supplementary material for: Somatic mutations as markers of outcome after azacitidine and allogeneic stem cell transplantation in higher-risk myelodysplastic syndromes
Source: Leukemia. 2018 Oct 5;33(3):785–90. doi: 10.1038/s41375-018-0284-9 (PMC6462855; doi:10.1038/s41375-018-0284-9)
Supplement: Supplementary file 1 — Supplementary text [file 41375_2018_284_MOESM1_ESM.docx]

**Supplementary Text**

**Definition of treatment outcomes**

Patients with MDS were considered responders if they achieved complete remission (CR, n=12), partial remission (PR, n=12) or haematological improvement (HI, n=6). Patients exhibiting stable disease (SD, n=23) or progressive disease (PD, n=12) were considered unresponsive.

Survival curves (OS and PFS) were estimated according to the Kaplan-Meier product-limit method and were tested for significant differences between groups using the log-rank test. PFS was defined as the time between the first dose of AZA and disease progression to AML in higher-risk MDS, and as date of relapse in AML following CR, or of death, whichever occurred first. Cox regression models were used in multivariate analysis for survival outcomes, after assessment of proportionality of hazards. All variables with a p-value less than 0.1 in the univariate regression were considered in the multivariate model with backward and stepwise conditional algorithm. In all analyses, 95% confidence intervals were reported for the main summary statistics, and all statistical comparisons were based on two-tailed tests, accepting p ≤0.05 as significant.

**NGS pipeline and Pyrosequencing**

Generated FASTQ sequencing files were then uploaded to SOPHiA DDM® platform, version four (SOPHiA GENETICS), that allows to detect, annotate and pre-classify genomic mutations (SNVs and Indels) through its artificial intelligence SOPHiA™. According to the SOPHiA pipeline, following adapter trimming and quality filtering, reads were aligned to the human reference genome (hg19 assembly). Variant calling of the resulted alignments was then performed using SOPHiA's in-house somatic variant caller, which takes into account background noise level at each region. The variant allelic fraction (VAF) (i.e., fraction of reads supporting the variant) was then calculated for each of the detected variants and VAF below the minimum were filtered (minimum 1% VAF for both SNP and INDEL). Moreover, detected variants were characterized by the effects on coding sequence, and scores such as pathogenicity were calculated.

After applying the SOPHiA DDM® platform filter- (a) Minimum VAF cut-off: 1% for SNP, 1% for INDEL, (b) Homopolymer length threshold: 10bp (c) Low coverage threshold: 1000x -, a manual filter was used to exclude known single nucleotide polymorphisms, variants localized in intronic and UTR regions and synonymous variants. Only exonic and splice sites variants were taken into consideration. Targeted-NGS sequencing data are stored at https://www.sophiagenetics.com (SOPHiA DDM platform), and can be extracted using the Sophia-DDM-v4 password-protected software. Raw data will be provided to Researchers upon request.

Changes in mutation burden were quantified in paired sample of BM and/or PB before and/or after four cycles of AZA by pyrosequencing, using specifically designed oligonucleotides targeting the mutated region (shown in supplementary Table 3). All mutations were confirmed and quantified in independent experiments. Reagents (PyroMark Gold Q96, QiagenSrl, Milan, Italy), instrumentation and software used for pyrosequencing analysis were as recommended by the manufacturers (PyroMark Q96 ID, DiatechPharmacogenetics, Jesi, Italy, PyroMark Assay Design and PyroMark Q24 version 2.0.6).

NGS data were confirmed using pyrosequencing assays: no significant differences in the VAF of specific mutations were observed by comparing the two techniques (supplementary Figure 2A). Moreover, VAF was similar in 23 paired BM-MNC and PB-WBC samples for most gene mutations, with the exception of those affecting IDH1 and IDH2, which were present at higher VAF in BM-MNC (supplementary Figure 2B). These data indicate that the mutation spectrum of PB-MNC may be representative of that of BM in most cases.

**Associations between mutations and patient characteristics.**

In our patients, DNMT3Amut and SRSF2mut were associated with a proportion of BM blasts greater than 20% (p=0.001 and p=0.026, respectively, Figure 1B). The proportion of IDH2mut and SRSF2mut clones directly correlated with BM-blasts (p=0.005 and p=0.007). As reported by others, TP53 mutations were significantly more frequent in patients with a complex karyotype (p<0.0001). Figure 1B reports significant associations between different mutations. Overall, patients with RUNX1 and ASXL1 mutations had a higher average number of mutations and of mutated genes, when compared to RUNX1 and ASXL1 wild-type (WT) patients (p=0.004 and p=0.0005, respectively, Figure 1B).
