# Supplementary material for: Somatic mutations as markers of outcome after azacitidine and allogeneic stem cell transplantation in higher-risk myelodysplastic syndromes
Source: Leukemia. 2018 Oct 5;33(3):785–90. doi: 10.1038/s41375-018-0284-9 (PMC6462855; doi:10.1038/s41375-018-0284-9)
Supplement: Supplementary file 2 — Supplementary Tables [file 41375_2018_284_MOESM2_ESM.docx]

**Supplementary Table 1**: Patient characteristics.

| **Patient Characteristics (n=65 patients)** | | |
| --- | --- | --- |
| **Age (median, range)** | | 59 (21-66) |
| **Gender (number)** | Female | 21 |
|  | Male | 44 |
| **BM-blasts % (median, range)** | | 15 (0-30) |
| **Blood counts (median, range)** | Hb (g/dl) | 9.4 (7.1-13.3) |
|  | Neutrophils (10^9^/L) | 0.6 (0.1-37) |
|  | Platelets (10^9^/L) | 75 (6-662) |
| **WHO Classification (n=65)** | MDS-EB-1 | 21 (32.3%) |
|  | MDS-EB-2 | 30 (46.1%) |
|  | MDS-U/ MDS-MLD | 2 (3.1%) |
|  | Low-blast count AML | 12 (18.5%) |
| **Karyotype (n=59)** | Normal | 23 (39.0%) |
|  | Trisomy 8 | 4 (6.8%) |
|  | -7 | 14 (23.7%) |
|  | Del 5q | 2 (3.4%) |
|  | Complex (≥3 abnormality) | 12 (20.3%) |
|  | Other | 4 (6.8%) |
| **IPSS (n=62)** | Low/Intermediate-1 | 2 (3.2%) |
|  | Intermediate-2 | 35 (56.5%) |
|  | High | 25 (40.3%) |
| **IPSS-R (n=49)** | Very low/Low | 3 (6.1%) |
|  | Intermediate | 8 (16.3%) |
|  | High | 14 (28.6%) |
|  | Very high | 24 (49.0%) |
| **AZA response (n=65)** | Complete remission | 12 (18.5%) |
|  | Partial response | 12 (18.5%) |
|  | Hematologic improvement | 6 (9.2%) |
|  | Stable disease | 23 (35.3%) |
|  | Progressive disease | 12 (18.5%) |
| **HSCT (n=65)** | Yes | 44 (67.7%) |
|  | Not | 21 (32.3%) |

**Supplementary table 2.** Comprehensive list of all genetic variants identified by NGS.

| **CHR** | **Genome_position** | **Gene** | **Exon_rank** | **c.DNA** | **protein** | **Ref** | **Alt** | **Type** | | **codingConsequence** | | **Sophia category** | **depth** | **var_percent** | **Sample_ID** |  |  |  |
| --- | --- | --- | --- | --- | --- | --- | --- | --- | --- | --- | --- | --- | --- | --- | --- | --- | --- | --- |
| 1 | 36937106 | CSF3R | 10 | c.1213G>A | p.Glu405Lys | C | T | SNP | | missense | | B | 9137 | 47,41 | 6277 |  |  |  |
| 18 | 42530168 | SETBP1 | 4 | c.863G>C | p.Gly288Ala | G | C | SNP | | missense | | B | 9979 | 1,84 | 6277 |  |  |  |
| 4 | 106157698 | TET2 | 3 | c.2599T>C | p.Tyr867His | T | C | SNP | | missense | | B | 12365 | 49,16 | 7721 |  |  |  |
| 4 | 106196834 | TET2 | 11 | c.5167C>T | p.Pro1723Ser | C | T | SNP | | missense | | C | 9940 | 49,52 | 7721 |  |  |  |
| 19 | 33792491 | CEBPA | 1 | c.830C>A | p.Ser277* | G | T | SNP | | nonsense | | A | 9706 | 38,25 | 7721 |  |  |  |
| 17 | 7578555 | TP53 | 5 | c.376-1G>A | p.? | C | T | SNP | | splice_acceptor_-1 | | A | 4926 | 65,94 | 7721 |  |  |  |
| 12 | 12022501 | ETV6 | 5 | c.613dupC | p.Leu205Profs*12 | TCCCCCC | TCCCCCCC | INDEL | | frameshift | | A | 16089 | 3,7 | 8391 |  |  |  |
| 2 | 198266831 | SF3B1 | 15 | c.2101G>T | p.Val701Phe | C | A | SNP | | missense | | B | 12725 | 3,23 | 8391 |  |  |  |
| 20 | 31022286 | ASXL1 | 12 | c.1772dupA | p.Tyr591*fs*1 | TA | TAA | INDEL | | frameshift | | A | 9811 | 16,98 | 8394 |  |  |  |
| 7 | 148543681 | EZH2 | 3 | c.126delT | p.Phe42Leufs*15 | TAAA | TAA | INDEL | | frameshift | | A | 8272 | 18,18 | 8394 |  |  |  |
| 7 | 148526936 | EZH2 | 5 | c.367delG | p.Glu123Lysfs*17 | TCC | TC | INDEL | | frameshift | | A | 8719 | 19,76 | 8394 |  |  |  |
| 1 | 36932463 | CSF3R | 17 | c.2087T>C | p.Met696Thr | A | G | SNP | | missense | | B | 12585 | 49,57 | 8394 |  |  |  |
| 21 | 36252852 | RUNX1 | 5 | c.508+2T>C | p.? | A | G | SNP | | splice_donor_+2 | | A | 12284 | 17,75 | 8394 |  |  |  |
| 2 | 209113113 | IDH1 | 4 | c.394C>T | p.Arg132Cys | G | A | SNP | | missense | | B | 5155 | 40,33 | 8398 |  |  |  |
| 17 | 74732959 | SRSF2 | 1 | c.284C>T | p.Pro95Leu | G | A | SNP | | missense | | B | 10675 | 32,86 | 8398 |  |  |  |
| X | 15836711 | ZRSR2 | 9 | c.773T>G | p.Val258Gly | T | G | SNP | | missense | | B | 5402 | 3,65 | 8398 |  |  |  |
| 2 | 25463507 | DNMT3A | 18 | c.2173+2T>C | p.? | A | G | SNP | | splice_donor_+2 | | A | 10452 | 38,49 | 8398 |  |  |  |
| 9 | 5081768 | JAK2 | 19 | c.2478G>T | p.Arg826Ser | G | T | SNP | | missense | | B | 7214 | 45,98 | 8399 |  |  |  |
| 21 | 36252877 | RUNX1 | 5 | c.485G>A | p.Arg162Lys | C | T | SNP | | missense | | B | 11970 | 75,1 | 8399 |  |  |  |
| 18 | 42533245 | SETBP1 | 4 | c.3940A>G | p.Ile1314Val | A | G | SNP | | missense | | B | 12157 | 45,63 | 8399 |  |  |  |
| 2 | 198267359 | SF3B1 | 14 | c.1998G>T | p.Lys666Asn | C | A | SNP | | missense | | A | 8132 | 45,59 | 8399 |  |  |  |
| 17 | 74732959 | SRSF2 | 1 | c.284C>T | p.Pro95Leu | G | A | SNP | | missense | | B | 9472 | 37,17 | 8399 |  |  |  |
| 12 | 112915480 | PTPN11 | 8 | c.879C>G | p.His293Gln | C | G | SNP | | missense | | B | 11977 | 50,27 | 8400 |  |  |  |
| 17 | 74732959 | SRSF2 | 1 | c.284C>A | p.Pro95His | G | T | SNP | | missense | | B | 11430 | 42,93 | 8400 |  |  |  |
| 15 | 90631934 | IDH2 | 4 | c.419G>A | p.Arg140Gln | C | T | SNP | | missense | | A | 10023 | 14,16 | 8401 |  |  |  |
| 12 | 112888138 | PTPN11 | 3 | c.154A>G | p.Thr52Ala | A | G | SNP | | missense | | B | 10183 | 2,93 | 8401 |  |  |  |
| 17 | 74732959 | SRSF2 | 1 | c.284C>T | p.Pro95Leu | G | A | SNP | | missense | | B | 9720 | 16,74 | 8401 |  |  |  |
| 20 | 31022367 | ASXL1 | 12 | c.1852A>T | p.Lys618* | A | T | SNP | | nonsense | | A | 8087 | 14,97 | 8401 |  |  |  |
| 21 | 36171612 | RUNX1 | 8 | c.952dupT | p.Ser318Phefs*? | GAAA | GAAAA | INDEL | | frameshift | | A | 12524 | 5,41 | 8411 |  |  |  |
| 2 | 209113113 | IDH1 | 4 | c.394C>T | p.Arg132Cys | G | A | SNP | | missense | | B | 6305 | 35,46 | 8411 |  |  |  |
| 21 | 36252865 | RUNX1 | 5 | c.497G>A | p.Arg166Gln | C | T | SNP | | missense | | B | 15772 | 8,8 | 8411 |  |  |  |
| 17 | 74732959 | SRSF2 | 1 | c.284C>G | p.Pro95Arg | G | C | SNP | | missense | | B | 12871 | 38,66 | 8411 |  |  |  |
| 17 | 7579344 | TP53 | 4 | c.342dupG | p.His115Alafs*34 | GC | GCC | INDEL | | frameshift | | A | 12683 | 3,19 | 8415 |  |  |  |
| 2 | 25468174 | DNMT3A | 13 | c.1502A>G | p.Asn501Ser | T | C | SNP | | missense | | B | 9626 | 46,15 | 8415 |  |  |  |
| 18 | 42530515 | SETBP1 | 4 | c.1210A>G | p.Ile404Val | A | G | SNP | | missense | | B | 10298 | 50,05 | 8415 |  |  |  |
| 4 | 106156097 | TET2 | 3 | c.998C>T | p.Pro333Leu | C | T | SNP | | missense | | B | 12975 | 50,16 | 8415 |  |  |  |
| 17 | 7578370 | TP53 | 5 | c.559+1G>A | p.? | C | T | SNP | | splice_donor_+1 | | A | 13084 | 2,22 | 8415 |  |  |  |
| 17 | 7578443 | TP53 | 5 | c.487T>C | p.Tyr163His | A | G | SNP | | missense | | B | 9764 | 21,62 | 8416 |  |  |  |
| 17 | 7577120 | TP53 | 8 | c.818G>A | p.Arg273His | C | T | SNP | | missense | | A | 10106 | 4,16 | 8416 |  |  |  |
| 4 | 106197606 | TET2 | 11 | c.5939C>T | p.Thr1980Ile | C | T | SNP | | missense | | B | 11496 | 48,99 | 8417 |  |  |  |
| X | 15833814 | ZRSR2 | 8 | c.572A>T | p.His191Leu | A | T | SNP | | missense | | B | 6111 | 20,77 | 8417 |  |  |  |
| 12 | 25398285 | KRAS | 2 | c.34G>A | p.Gly12Ser | C | T | SNP | | missense | | A | 11452 | 2,66 | 8419 |  |  |  |
| 1 | 115258747 | NRAS | 2 | c.35G>T | p.Gly12Val | C | A | SNP | | missense | | A | 13265 | 3,61 | 8419 |  |  |  |
| 20 | 31022885 | ASXL1 | 12 | c.2372_2373dupCT | p.Gly792Leufs*27 | G | GTC | INDEL | | frameshift | | A | 12342 | 26,13 | 8420 |  |  |  |
| 2 | 25457242 | DNMT3A | 23 | c.2645G>A | p.Arg882His | C | T | SNP | | missense | | B | 13373 | 4,34 | 8420 |  |  |  |
| 21 | 36252861 | RUNX1 | 5 | c.501T>A | p.Ser167Arg | A | T | SNP | | missense | | B | 12645 | 2,85 | 8420 |  |  |  |
| 17 | 74732959 | SRSF2 | 1 | c.284C>T | p.Pro95Leu | G | A | SNP | | missense | | B | 10229 | 27,14 | 8420 |  |  |  |
| 17 | 7578412 | TP53 | 5 | c.518T>C | p.Val173Ala | A | G | SNP | | missense | | B | 12566 | 1,79 | 8420 |  |  |  |
| 17 | 7574034 | TP53 | 10 | c.994-1G>A | p.? | C | T | SNP | | splice_acceptor_-1 | | A | 9928 | 1,38 | 8420 |  |  |  |
| 21 | 36231768 | RUNX1 | 6 | c.613+1_613+2delGT | p.? | TAC | T | INDEL | | splice_donor_indel | | A | 10418 | 29,94 | 8420 |  |  |  |
| 20 | 31022898 | ASXL1 | 12 | c.2385delC | p.Trp796Glyfs*22 | TCC | TC | INDEL | | frameshift | | A | 11694 | 40,35 | 8421 |  |  |  |
| 13 | 28608273 | FLT3 | 14 | c.1747_1782dup | p.Gly583_Phe594dup | T | TGAAATCAACGTAGAAGTA  CTCATTATCTGAGGAGCC | INDEL | | inframe_36 | | A | 12904 | 4 | 8421 |  |  |  |
| 13 | 28608235 | FLT3 | 14 | c.1746_1820dup | p.Pro606_Arg607ins25 | T | TCTTGGAAACTCCCATTTG  AGATCATATTCATATTCTC  TGAAATCAACGTAGAAGTAC  TCATTATCTGAGGAGCCG | INDEL | | inframe_75 | | A | 13705 | 15 | 8421 |  |  |  |
| 13 | 28592623 | FLT3 | 20 | c.2522A>T | p.Asn841Ile | T | A | SNP | | missense | | B | 11109 | 1,57 | 8421 |  |  |  |
| 18 | 42531913 | SETBP1 | 4 | c.2608G>A | p.Gly870Ser | G | A | SNP | | missense | | A | 9266 | 41,4 | 8421 |  |  |  |
| 21 | 36164689 | RUNX1 | 9 | c.1182_1185dupCCCG | p.Phe396Profs*? | A | ACGGG | INDEL | | frameshift | | A | 8841 | 19,58 | 8424 |  |  |  |
| 19 | 33792731 | CEBPA | 1 | c.584_589dupACCCGC | p.His195_Pro196dup | G | GGCGGGT | INDEL | | inframe_6 | | A | 6836 | 31,76 | 8424 |  |  |  |
| 19 | 33792753 | CEBPA | 1 | c.568T>C | p.Ser190Pro | A | G | SNP | | missense | | B | 6416 | 2,79 | 8424 |  |  |  |
| 1 | 115258748 | NRAS | 2 | c.34G>A | p.Gly12Ser | C | T | SNP | | missense | | A | 10814 | 10,64 | 8424 |  |  |  |
| 18 | 42530225 | SETBP1 | 4 | c.920A>G | p.Glu307Gly | A | G | SNP | | missense | | B | 9465 | 1,38 | 8424 |  |  |  |
| 4 | 106158560 | TET2 | 3 | c.3461A>G | p.Asn1154Ser | A | G | SNP | | missense | | B | 8163 | 50,07 | 8424 |  |  |  |
| 21 | 36164831 | RUNX1 | 9 | c.1040_1043dupTGCA | p.Tyr349Alafs*? | G | GTGCA | INDEL | | frameshift | | A | 9289 | 25,63 | 8425 |  |  |  |
| 17 | 7574013 | TP53 | 10 | c.1013delT | p.Phe338Serfs*7 | GAA | GA | INDEL | | frameshift | | A | 8581 | 2,21 | 8425 |  |  |  |
| 17 | 7578401 | TP53 | 5 | c.529C>T | p.Pro177Ser | G | A | SNP | | missense | | B | 10103 | 30,34 | 8425 |  |  |  |
| 17 | 7578190 | TP53 | 6 | c.659A>G | p.Tyr220Cys | T | C | SNP | | missense | | A | 9622 | 32,61 | 8425 |  |  |  |
| 11 | 119148880 | CBL | 8 | c.1100A>C | p.Gln367Pro | A | C | SNP | | missense | | A | 7738 | 35,28 | 8426 |  |  |  |
| 21 | 36231791 | RUNX1 | 6 | c.593A>G | p.Asp198Gly | T | C | SNP | | missense | | B | 7602 | 42,33 | 8426 |  |  |  |
| 18 | 42531907 | SETBP1 | 4 | c.2602G>A | p.Asp868Asn | G | A | SNP | | missense | | A | 6800 | 42,88 | 8426 |  |  |  |
| 18 | 42532604 | SETBP1 | 4 | c.3299A>G | p.His1100Arg | A | G | SNP | | missense | | B | 11837 | 52,37 | 8428 |  |  |  |
| 2 | 198266834 | SF3B1 | 15 | c.2098A>G | p.Lys700Glu | T | C | SNP | | missense | | B | 9354 | 7,45 | 8428 |  |  |  |
| 20 | 31022826 | ASXL1 | 12 | c.2315_2357del | p.Ala772Glufs*32 | GTAGCTGAGAGATTAGT  GGAGCAGCCTCAGTTGCAT  CCGGATGT | G | INDEL | | frameshift | | A | 10193 | 19 | 8429 |  |  |  |
| 11 | 119148973 | CBL | 8 | c.1193A>G | p.His398Arg | A | G | SNP | | missense | | B | 11116 | 2,17 | 8429 |  |  |  |
| 15 | 90631934 | IDH2 | 4 | c.419G>A | p.Arg140Gln | C | T | SNP | | missense | | A | 11110 | 28,12 | 8429 |  |  |  |
| 9 | 5073770 | JAK2 | 14 | c.1849G>T | p.Val617Phe | G | T | SNP | | missense | | A | 8834 | 25,61 | 8429 |  |  |  |
| 21 | 44514777 | U2AF1 | 6 | c.470A>C | p.Gln157Pro | T | G | SNP | | missense | | B | 13416 | 32,4 | 8429 |  |  |  |
| X | 15827389 | ZRSR2 | 7 | c.505C>T | p.Arg169* | C | T | SNP | | nonsense | | A | 5413 | 55,2 | 8429 |  |  |  |
| 20 | 31022402 | ASXL1 | 12 | c.1900_1922del | p.Glu635Argfs*15 | TCACCACTGCCATAGAG  AGGCGGC | T | INDEL | | frameshift | | A | 6251 | 50 | 8430 |  |  |  |
| 13 | 28608237 | FLT3 | 14 | c.1795_1818dup | p.Tyr599_Pro606dup | T | TTGGAAACTCCCATTT  GAGATCATA | INDEL | | inframe_24 | | A | 9228 | 27 | 8430 |  |  |  |
| 18 | 42531907 | SETBP1 | 4 | c.2602G>A | p.Asp868Asn | G | A | SNP | | missense | | A | 6610 | 47,49 | 8430 |  |  |  |
| 12 | 112888202 | PTPN11 | 3 | c.218C>T | p.Thr73Ile | C | T | SNP | | missense | | A | 10191 | 1,21 | 8431 |  |  |  |
| 1 | 36932254 | CSF3R | 17 | c.2215C>T | p.Gln739* | G | A | SNP | | nonsense | | A | 8750 | 6,1 | 8431 |  |  |  |
| 1 | 36932224 | CSF3R | 17 | c.2245C>T | p.Gln749* | G | A | SNP | | nonsense | | A | 9026 | 2,35 | 8431 |  |  |  |
| 19 | 33793252 | CEBPA | 1 | c.68delC | p.Pro23Argfs*137 | CGGGGGG | CGGGGG | INDEL | | frameshift | | A | 14978 | 1,78 | 8432 |  |  |  |
| 4 | 106197235 | TET2 | 11 | c.5570delC | p.Pro1857Leufs*30 | TCC | TC | INDEL | | frameshift | | A | 13717 | 41,61 | 8432 |  |  |  |
| 20 | 31022938 | ASXL1 | 12 | c.2423C>A | p.Pro808His | C | A | SNP | | missense | | B | 11910 | 40,24 | 8432 |  |  |  |
| 19 | 33793259 | CEBPA | 1 | c.62G>A | p.Ser21Asn | C | T | SNP | | missense | | B | 14162 | 1,89 | 8432 |  |  |  |
| 17 | 74732959 | SRSF2 | 1 | c.284C>A | p.Pro95His | G | T | SNP | | missense | | B | 13096 | 41,66 | 8432 |  |  |  |
| 20 | 31022853 | ASXL1 | 12 | c.2338C>T | p.Gln780* | C | T | SNP | | nonsense | | A | 12817 | 37,97 | 8432 |  |  |  |
| 2 | 25471070 | DNMT3A | 7 | c.691C>T | p.Gln231* | G | A | SNP | | nonsense | | A | 12513 | 3,64 | 8433 |  |  |  |
| 17 | 7577022 | TP53 | 8 | c.916C>T | p.Arg306* | G | A | SNP | | nonsense | | A | 12540 | 6,94 | 8433 |  |  |  |
| 20 | 31022547 | ASXL1 | 12 | c.2036dupG | p.Gly680Argfs*38 | AGGGG | AGGGGG | INDEL | | frameshift | | A | 9327 | 20,16 | 8434 |  |  |  |
| 7 | 148526825 | EZH2 | 5 | c.478delG | p.Asp160Ilefs*7 | TCCCC | TCCC | INDEL | | frameshift | | A | 7041 | 9 | 8434 |  |  |  |
| 21 | 44524456 | U2AF1 | 2 | c.101C>A | p.Ser34Tyr | G | T | SNP | | missense | | B | 11637 | 22,19 | 8434 |  |  |  |
| 19 | 33792864 | CEBPA | 1 | c.449_456dupTCGGGGCG | p.Pro153Serfs*10 | G | GCGCCCCGA | INDEL | | frameshift | | A | 11278 | 4,1 | 8435 |  |  |  |
| 17 | 74732959 | SRSF2 | 1 | c.284C>G | p.Pro95Arg | G | C | SNP | | missense | | B | 11771 | 43,16 | 8435 |  |  |  |
| 2 | 198266834 | SF3B1 | 15 | c.2098A>G | p.Lys700Glu | T | C | SNP | | missense | | B | 6474 | 2,59 | 8436 |  |  |  |
| 17 | 7577108 | TP53 | 8 | c.830G>A | p.Cys277Tyr | C | T | SNP | | missense | | B | 7197 | 19,79 | 8436 |  |  |  |
| 4 | 106164914 | TET2 | 6 | c.3782G>A | p.Arg1261His | G | A | SNP | | missense | | B | 8702 | 31,3 | 8437 |  |  |  |
| X | 15818028 | ZRSR2 | 3 | c.155T>G | p.Phe52Cys | T | G | SNP | | missense | | B | 2214 | 96,93 | 8437 |  |  |  |
| 4 | 106182914 | TET2 | 8 | c.3955-2A>G | p.? | A | G | SNP | | splice_acceptor_-2 | | A | 6355 | 45,55 | 8437 |  |  |  |
| X | 15836766 | ZRSR2 | 9 | c.827+2dupT | p.? | GT | GTT | INDEL | | splice_donor_indel | | A | 3862 | 82,06 | 8437 |  |  |  |
| 1 | 36932503 | CSF3R | 17 | c.2047G>A | p.Gly683Arg | C | T | SNP | | missense | | C | 10193 | 48,81 | 8438 |  |  |  |
| 5 | 170837543 | NPM1 | 11 | c.860_863dupTCTG | p.Trp288Cysfs*? | C | CTCTG | INDEL | | frameshift | | A | 5913 | 15,49 | 8467 |  |  |  |
| 2 | 25457242 | DNMT3A | 23 | c.2645G>A | p.Arg882His | C | T | SNP | | missense | | B | 10832 | 18,78 | 8467 |  |  |  |
| 2 | 25470588 | DNMT3A | 8 | c.886G>A | p.Val296Met | C | T | SNP | | missense | | B | 8533 | 1,28 | 8467 |  |  |  |
| 1 | 36932047 | CSF3R | 17 | c.2422G>A | p.Glu808Lys | C | T | SNP | | missense | | B | 10681 | 47,46 | 8468 |  |  |  |
| 21 | 36164685 | RUNX1 | 9 | c.1189delC | p.Gln397Lysfs*? | TGG | TG | INDEL | | frameshift | | A | 8734 | 33,86 | 8470 |  |  |  |
| 2 | 25457242 | DNMT3A | 23 | c.2645G>A | p.Arg882His | C | T | SNP | | missense | | B | 11148 | 46,39 | 8470 |  |  |  |
| 13 | 28592642 | FLT3 | 20 | c.2503G>T | p.Asp835Tyr | C | A | SNP | | missense | | A | 9749 | 1,07 | 8470 |  |  |  |
| 21 | 36231782 | RUNX1 | 6 | c.602G>A | p.Arg201Gln | C | T | SNP | | missense | | A | 8506 | 3,73 | 8470 |  |  |  |
| 17 | 74732959 | SRSF2 | 1 | c.284C>T | p.Pro95Leu | G | A | SNP | | missense | | B | 8998 | 45,73 | 8470 |  |  |  |
| 4 | 106156163 | TET2 | 3 | c.1064G>A | p.Gly355Asp | G | A | SNP | | missense | | C | 12462 | 50,31 | 8470 |  |  |  |
| 21 | 36171718 | RUNX1 | 8 | c.847C>T | p.Gln283* | G | A | SNP | | nonsense | | A | 9053 | 3,1 | 8470 |  |  |  |
| 4 | 106196237 | TET2 | 11 | c.4570C>T | p.Gln1524* | C | T | SNP | | nonsense | | A | 8924 | 47,37 | 8470 |  |  |  |
| 4 | 106196560 | TET2 | 11 | c.4893T>A | p.Tyr1631* | T | A | SNP | | nonsense | | A | 9455 | 46,97 | 8470 |  |  |  |
| 21 | 36206885 | RUNX1 | 7 | c.620_626delGGCAGAA | p.Arg207Hisfs*2 | TTTCTGCC | T | INDEL | | frameshift | | A | 10322 | 9,95 | 8473 |  |  |  |
| 1 | 36939403 | CSF3R | 5 | c.447G>C | p.Glu149Asp | C | G | SNP | | missense | | B | 12860 | 46,32 | 8473 |  |  |  |
| 18 | 42530225 | SETBP1 | 4 | c.920A>G | p.Glu307Gly | A | G | SNP | | missense | | B | 11053 | 1,99 | 8473 |  |  |  |
| 21 | 44524456 | U2AF1 | 2 | c.101C>T | p.Ser34Phe | G | A | SNP | | missense | | B | 13506 | 31,49 | 8473 |  |  |  |
| 20 | 31022832 | ASXL1 | 12 | c.2317G>T | p.Glu773* | G | T | SNP | | nonsense | | A | 12457 | 21,93 | 8473 |  |  |  |
| 21 | 36259324 | RUNX1 | 4 | c.167T>C | p.Leu56Ser | A | G | SNP | | missense | | B | 10777 | 48,24 | 8474 |  |  |  |
| 17 | 7578445 | TP53 | 5 | c.485T>A | p.Ile162Asn | A | T | SNP | | missense | | B | 11916 | 46,79 | 8474 |  |  |  |
| 17 | 7577556 | TP53 | 7 | c.725G>A | p.Cys242Tyr | C | T | SNP | | missense | | A | 9741 | 45,02 | 8474 |  |  |  |
| 4 | 106156613 | TET2 | 3 | c.1516delA | p.Arg506Aspfs*27 | CAA | CA | INDEL | | frameshift | | A | 12246 | 87,97 | 8476 |  |  |  |
| 2 | 25463289 | DNMT3A | 19 | c.2204A>G | p.Tyr735Cys | T | C | SNP | | missense | | B | 11295 | 44,57 | 8476 |  |  |  |
| 2 | 25457243 | DNMT3A | 23 | c.2644C>T | p.Arg882Cys | G | A | SNP | | missense | | B | 12687 | 41,36 | 8486 |  |  |  |
| 1 | 115258748 | NRAS | 2 | c.34G>A | p.Gly12Ser | C | T | SNP | | missense | | A | 11852 | 32,97 | 8486 |  |  |  |
| 1 | 115258744 | NRAS | 2 | c.38G>A | p.Gly13Asp | C | T | SNP | | missense | | A | 11887 | 1,81 | 8486 |  |  |  |
| 12 | 112888198 | PTPN11 | 3 | c.214G>A | p.Ala72Thr | G | A | SNP | | missense | | B | 13542 | 3,38 | 8486 |  |  |  |
| 18 | 42531913 | SETBP1 | 4 | c.2608G>A | p.Gly870Ser | G | A | SNP | | missense | | A | 9250 | 4,04 | 8486 |  |  |  |
| 1 | 36932209 | CSF3R | 17 | c.2260C>T | p.Gln754* | G | A | SNP | | nonsense | | A | 12035 | 35,26 | 8486 |  |  |  |
| 20 | 31023321 | ASXL1 | 12 | c.2810delC | p.Pro937Leufs*8 | ACCCC | ACCC | INDEL | | frameshift | | A | 11429 | 42,23 | 8487 |  |  |  |
| 1 | 115258747 | NRAS | 2 | c.35G>A | p.Gly12Asp | C | T | SNP | | missense | | A | 10023 | 34,41 | 8487 |  |  |  |
| 4 | 106164860 | TET2 | 6 | c.3728A>G | p.Lys1243Arg | A | G | SNP | | missense | | B | 11437 | 49 | 8487 |  |  |  |
| 4 | 106164914 | TET2 | 6 | c.3782G>A | p.Arg1261His | G | A | SNP | | missense | | B | 10601 | 44,55 | 8487 |  |  |  |
| 4 | 106182940 | TET2 | 8 | c.3979C>T | p.Gln1327* | C | T | SNP | | nonsense | | A | 7282 | 42,6 | 8487 |  |  |  |
| 20 | 31022837 | ASXL1 | 12 | c.2324delT | p.Leu775*fs*1 | ATT | AT | INDEL | | frameshift | | A | 10983 | 10,84 | 8488 |  |  |  |
| 21 | 36206787 | RUNX1 | 7 | c.724delC | p.His242Thrfs*12 | TGG | TG | INDEL | | frameshift | | A | 12145 | 12,17 | 8488 |  |  |  |
| 2 | 209113113 | IDH1 | 4 | c.394C>T | p.Arg132Cys | G | A | SNP | | missense | | B | 5159 | 7 | 8488 |  |  |  |
| 15 | 90631934 | IDH2 | 4 | c.419G>A | p.Arg140Gln | C | T | SNP | | missense | | A | 10583 | 1,31 | 8488 |  |  |  |
| 7 | 148543657 | EZH2 | 3 | c.151G>T | p.Glu51* | C | A | SNP | | nonsense | | A | 7207 | 12,54 | 8488 |  |  |  |
| 20 | 31022363 | ASXL1 | 12 | c.1849dupA | p.Ile617Asnfs*2 | CA | CAA | INDEL | | frameshift | | A | 10797 | 39,67 | 8489 |  |  |  |
| 12 | 25380255 | KRAS | 3 | c.203G>T | p.Arg68Met | C | A | SNP | | missense | | B | 10670 | 4,93 | 8489 |  |  |  |
| 12 | 112888165 | PTPN11 | 3 | c.181G>A | p.Asp61Asn | G | A | SNP | | missense | | A | 14113 | 35,4 | 8489 |  |  |  |
| 11 | 119149251 | CBL | 9 | c.1259G>A | p.Arg420Gln | G | A | SNP | | missense | | A | 8901 | 60,57 | 8490 |  |  |  |
| 1 | 36935408 | CSF3R | 11 | c.1319G>A | p.Arg440Gln | C | T | SNP | | missense | | B | 8432 | 49,79 | 8490 |  |  |  |
| 2 | 198266713 | SF3B1 | 15 | c.2219G>A | p.Gly740Glu | C | T | SNP | | missense | | B | 10667 | 12,19 | 8490 |  |  |  |
| 21 | 44524456 | U2AF1 | 2 | c.101C>A | p.Ser34Tyr | G | T | SNP | | missense | | B | 11327 | 44,16 | 8490 |  |  |  |
| 20 | 31022847 | ASXL1 | 12 | c.2332C>T | p.Gln778* | C | T | SNP | | nonsense | | A | 10384 | 27,2 | 8490 |  |  |  |
| 21 | 36252853 | RUNX1 | 5 | c.508+1G>T | p.? | C | A | SNP | | splice_donor_+1 | | A | 12449 | 1,51 | 8490 |  |  |  |
| 21 | 36259324 | RUNX1 | 4 | c.167T>C | p.Leu56Ser | A | G | SNP | | missense | | B | 9795 | 48,31 | 8496 |  |  |  |
| 17 | 7578550 | TP53 | 5 | c.380C>T | p.Ser127Phe | G | A | SNP | | missense | | B | 8493 | 92,57 | 8496 |  |  |  |
| 4 | 106157713 | TET2 | 3 | c.2615dupT | p.Ile873Aspfs*28 | GT | GTT | INDEL | | frameshift | | A | 13788 | 37,68 | 8531 |  |  |  |
| 13 | 28608473 | FLT3 | 13 | c.1669G>A | p.Val557Ile | C | T | SNP | | missense | | D | 14653 | 46,43 | 8531 |  |  |  |
| 4 | 106157698 | TET2 | 3 | c.2599T>C | p.Tyr867His | T | C | SNP | | missense | | B | 13465 | 45,63 | 8531 |  |  |  |
| 4 | 106196834 | TET2 | 11 | c.5167C>T | p.Pro1723Ser | C | T | SNP | | missense | | C | 12889 | 48,26 | 8531 |  |  |  |
| 4 | 106162498 | TET2 | 4 | c.3412C>T | p.Gln1138* | C | T | SNP | | nonsense | | A | 9783 | 40,5 | 8531 |  |  |  |
| X | 15841258 | ZRSR2 | 11 | c.1344_1346delCAG | p.Arg449del | CGCA | C | INDEL | | inframe_3 | | B | 10800 | 1,19 | 8563 |  |  |  |
| X | 15841230 | ZRSR2 | 11 | c.1338_1343dupGAGCCG | p.Ser447_Arg448dup | C | CAGCCGG | INDEL | | inframe_6 | | A | 11041 | 31,41 | 8563 |  |  |  |
| 20 | 31023821 | ASXL1 | 12 | c.3306G>T | p.Glu1102Asp | G | T | SNP | | missense | | B | 14367 | 51,49 | 8563 |  |  |  |
| 18 | 42530210 | SETBP1 | 4 | c.905C>T | p.Pro302Leu | C | T | SNP | | missense | | B | 10812 | 1,09 | 8563 |  |  |  |
| 17 | 7577559 | TP53 | 7 | c.722C>A | p.Ser241Tyr | G | T | SNP | | missense | | B | 5738 | 70,43 | 8563 |  |  |  |
| 20 | 31022658 | ASXL1 | 12 | c.2147_2153delCTAGGAG | p.Ala716Glufs*7 | AGAGCTAG | A | INDEL | | frameshift | | A | 11331 | 18,19 | 8749 |  |  |  |
| 2 | 25523096 | DNMT3A | 3 | c.89A>C | p.Glu30Ala | T | G | SNP | | missense | | B | 10300 | 52,36 | 8749 |  |  |  |
| 4 | 106156963 | TET2 | 3 | c.1864C>T | p.Gln622* | C | T | SNP | | nonsense | | A | 12401 | 3,83 | 8749 |  |  |  |
| 11 | 32417942 | WT1 | 7 | c.1109_1110insCC | p.Val371Leufs*5 | A | AGG | INDEL | | frameshift | | A | 7245 | 14,58 | 8750 |  |  |  |
| 13 | 28608291 | FLT3 | 14 | c.1765T>C | p.Tyr589His | A | G | SNP | | missense | | B | 9385 | 4,34 | 8750 |  |  |  |
| 21 | 36259324 | RUNX1 | 4 | c.167T>C | p.Leu56Ser | A | G | SNP | | missense | | B | 6543 | 45,99 | 8750 |  |  |  |
| 21 | 36259172 | RUNX1 | 4 | c.319C>T | p.Arg107Cys | G | A | SNP | | missense | | B | 6967 | 14,58 | 8750 |  |  |  |
| 11 | 32417943 | WT1 | 7 | c.1109G>C | p.Arg370Pro | C | G | SNP | | missense | | B | 7011 | 15,56 | 8750 |  |  |  |
| 2 | 198266834 | SF3B1 | 15 | c.2098A>G | p.Lys700Glu | T | C | SNP | | missense | | B | 10579 | 14,74 | 8751 |  |  |  |
| 18 | 42530327 | SETBP1 | 4 | c.1022A>G | p.Asp341Gly | A | G | SNP | | missense | | B | 12186 | 46,57 | 8753 |  |  |  |
| 21 | 44524456 | U2AF1 | 2 | c.101C>T | p.Ser34Phe | G | A | SNP | | missense | | B | 14150 | 34,98 | 8753 |  |  |  |
| 20 | 31022418 | ASXL1 | 12 | c.1903G>T | p.Glu635* | G | T | SNP | | nonsense | | A | 9583 | 9,76 | 8753 |  |  |  |
| 17 | 7578175 | TP53 | 6 | c.672+2T>A | p.? | A | T | SNP | | splice_donor_+2 | | A | 7662 | 59,85 | 8753 |  |  |  |
| 20 | 31022572 | ASXL1 | 12 | c.2060_2061delGT | p.Cys687Tyrfs*30 | AGT | A | INDEL | | frameshift | | A | 8861 | 2,97 | 8757 |  |  |  |
| 1 | 36935408 | CSF3R | 11 | c.1319G>A | p.Arg440Gln | C | T | SNP | | missense | | B | 8637 | 48,96 | 8757 |  |  |  |
| 4 | 106157926 | TET2 | 3 | c.2827C>T | p.Gln943* | C | T | SNP | | nonsense | | A | 11282 | 2,68 | 8757 |  |  |  |
| 20 | 31024310 | ASXL1 | 12 | c.3796delC | p.Thr1267Leufs*13 | TC | T | INDEL | | frameshift | | A | 13377 | 32,6 | 8760 |  |  |  |
| 21 | 36259198 | RUNX1 | 4 | c.292delC | p.Leu98Serfs*24 | AGG | AG | INDEL | | frameshift | | A | 11603 | 40,08 | 8760 |  |  |  |
| 20 | 31024314 | ASXL1 | 12 | c.3799A>T | p.Thr1267Ser | A | T | SNP | | missense | | B | 12923 | 33,77 | 8760 |  |  |  |
| 20 | 31022981 | ASXL1 | 12 | c.2468delT | p.Leu823*fs*1 | ATT | AT | INDEL | | frameshift | | A | 12646 | 26,85 | 8818 |  |  |  |
| 20 | 31023472 | ASXL1 | 12 | c.2957A>G | p.Asn986Ser | A | G | SNP | | missense | | B | 13865 | 50,36 | 8818 |  |  |  |
| 2 | 25457270 | DNMT3A | 23 | c.2617C>A | p.His873Asn | G | T | SNP | | missense | | B | 14593 | 33,92 | 8818 |  |  |  |
| 15 | 90631838 | IDH2 | 4 | c.515G>A | p.Arg172Lys | C | T | SNP | | missense | | B | 13393 | 29,4 | 8818 |  |  |  |
| 12 | 112919923 | PTPN11 | 10 | c.1138T>C | p.Tyr380His | T | C | SNP | | missense | | B | 13904 | 49,37 | 8818 |  |  |  |
| 21 | 36259195 | RUNX1 | 4 | c.296G>A | p.Cys99Tyr | C | T | SNP | | missense | | B | 12751 | 2,24 | 8818 |  |  |  |
| X | 15836709 | ZRSR2 | 9 | c.772-1G>T | p.? | G | T | SNP | | splice_acceptor_-1 | | A | 5660 | 65,39 | 8818 |  |  |  |
| 2 | 25458696 | DNMT3A | 22 | c.2479-2A>C | p.? | T | G | SNP | | splice_acceptor_-2 | | A | 13138 | 30,68 | 8818 |  |  |  |
| 18 | 42533261 | SETBP1 | 4 | c.3956T>C | p.Met1319Thr | T | C | SNP | | missense | | B | 10252 | 48,3 | 8819 |  |  |  |
| 2 | 25457242 | DNMT3A | 23 | c.2645G>A | p.Arg882His | C | T | SNP | | missense | | B | 11078 | 45,74 | 8822 |  |  |  |
| 15 | 90631934 | IDH2 | 4 | c.419G>A | p.Arg140Gln | C | T | SNP | | missense | | A | 8895 | 47,77 | 8822 |  |  |  |
| 17 | 74732959 | SRSF2 | 1 | c.284C>G | p.Pro95Arg | G | C | SNP | | missense | | B | 6014 | 46,08 | 8822 |  |  |  |
| 20 | 31022402 | ASXL1 | 12 | c.1900_1922del | p.Glu635Argfs*15 | TCACCACTGCCATA  GAGAGGCGGC | T | INDEL | | frameshift | | A | 8860 | 46 | 8824 |  |  |  |
| 21 | 36164863 | RUNX1 | 9 | c.1011dupC | p.Ala338Argfs*? | CGGGG | CGGGGG | INDEL | | frameshift | | A | 11874 | 25,16 | 8824 |  |  |  |
| 21 | 36259334 | RUNX1 | 4 | c.156_157insTT | p.Ser53Leufs*20 | T | TAA | INDEL | | frameshift | | A | 11001 | 6,46 | 8824 |  |  |  |
| 1 | 36932463 | CSF3R | 17 | c.2087T>C | p.Met696Thr | A | G | SNP | | missense | | B | 12746 | 51,38 | 8824 |  |  |  |
| 1 | 115256475 | NRAS | 3 | c.236T>G | p.Leu79Arg | A | C | SNP | | missense | | B | 9820 | 45,43 | 8824 |  |  |  |
| 1 | 115258747 | NRAS | 2 | c.35G>T | p.Gly12Val | C | A | SNP | | missense | | A | 11304 | 42,19 | 8824 |  |  |  |
| 21 | 36259335 | RUNX1 | 4 | c.156G>C | p.Met52Ile | C | G | SNP | | missense | | B | 10754 | 6,6 | 8824 |  |  |  |
| 7 | 148543568 | EZH2 | 3 | c.233_239delGCGGGAC | p.Arg78Leufs*6 | AGTCCCGC | A | INDEL | | frameshift | | A | 7521 | 20,45 | 8825 |  |  |  |
| 19 | 33792731 | CEBPA | 1 | c.584_589dupACCCGC | p.His195_Pro196dup | G | GGCGGGT | INDEL | | inframe_6 | | A | 6696 | 29,7 | 8825 |  |  |  |
| 19 | 33792753 | CEBPA | 1 | c.568T>C | p.Ser190Pro | A | G | SNP | | missense | | B | 6389 | 2,54 | 8825 |  |  |  |
| 15 | 90631838 | IDH2 | 4 | c.515G>A | p.Arg172Lys | C | T | SNP | | missense | | B | 10095 | 9,86 | 8825 |  |  |  |
| 12 | 112926888 | PTPN11 | 13 | c.1508G>A | p.Gly503Glu | G | A | SNP | | missense | | A | 12053 | 16,65 | 8825 |  |  |  |
| 21 | 36252941 | RUNX1 | 5 | c.421T>C | p.Ser141Pro | A | G | SNP | | missense | | B | 10268 | 16,49 | 8825 |  |  |  |
| 12 | 11992215 | ETV6 | 3 | c.305_306insCGGATTTCA  GGCCGTAG | p.Arg103Glyfs*25 | T | TCGGATTTCAGGCCGTAG | INDEL | | frameshift | | A | 13188 | 3 | 8977 |  |  |  |
| 11 | 32417914 | WT1 | 7 | c.1137_1138insG | p.Arg380Alafs*5 | G | GC | INDEL | | frameshift | | A | 12682 | 7,06 | 8977 |  |  |  |
| 20 | 31022625 | ASXL1 | 12 | c.2110G>A | p.Gly704Arg | G | A | SNP | | missense | | B | 11233 | 48,1 | 8977 |  |  |  |
| 12 | 25380275 | KRAS | 3 | c.183A>C | p.Gln61His | T | G | SNP | | missense | | A | 11937 | 7,77 | 8977 |  |  |  |
| 11 | 32417873 | WT1 | 7 | c.1179T>A | p.Cys393* | A | T | SNP | | nonsense | | A | 13126 | 2,3 | 8977 |  |  |  |
| X | 15841258 | ZRSR2 | 11 | c.1344_1346delCAG | p.Arg449del | CGCA | C | INDEL | | inframe_3 | | B | 6323 | 2,59 | 8984 |  |  |  |
| X | 15841230 | ZRSR2 | 11 | c.1338_1343dupGAGCCG | p.Ser447_Arg448dup | C | CAGCCGG | INDEL | | inframe_6 | | A | 6726 | 56,5 | 8984 |  |  |  |
| 20 | 31024797 | ASXL1 | 12 | c.4282T>C | p.Ser1428Pro | T | C | SNP | | missense | | B | 13174 | 46,33 | 8984 |  |  |  |
| 2 | 198267369 | SF3B1 | 14 | c.1988C>T | p.Thr663Ile | G | A | SNP | | missense | | B | 10936 | 1,34 | 8984 |  |  |  |
| 2 | 198266834 | SF3B1 | 15 | c.2098A>G | p.Lys700Glu | T | C | SNP | | missense | | B | 11975 | 27,11 | 8984 |  |  |  |
| 2 | 25457242 | DNMT3A | 23 | c.2645G>A | p.Arg882His | C | T | SNP | | missense | | B | 11113 | 34,98 | 8985 |  |  |  |
| 7 | 148516722 | EZH2 | 9 | c.965A>G | p.Asn322Ser | T | C | SNP | | missense | | B | 8499 | 35,39 | 8985 |  |  |  |
| 15 | 90631838 | IDH2 | 4 | c.515G>A | p.Arg172Lys | C | T | SNP | | missense | | B | 9964 | 21,01 | 8985 |  |  |  |
| 4 | 106190797 | TET2 | 9 | c.4075C>A | p.Arg1359Ser | C | A | SNP | | missense | | B | 10906 | 3,28 | 8985 |  |  |  |
| 7 | 148506483 | EZH2 | 18 | c.2030-1G>C | p.? | C | G | SNP | | splice_acceptor_-1 | | A | 6663 | 1,01 | 8985 |  |  |  |
| 20 | 31022952 | ASXL1 | 12 | c.2439_2449delCATTCTGTCTC | p.Ile814Serfs*4 | CCCATTCTGTCT | C | INDEL | | frameshift | | A | 8806 | 47 | 8986 |  |  |  |
| 19 | 33793179 | CEBPA | 1 | c.141delC | p.Ala48Profs*112 | CGG | CG | INDEL | | frameshift | | A | 8804 | 39,28 | 8986 |  |  |  |
| X | 15841230 | ZRSR2 | 11 | c.1338_1343dupGAGCCG | p.Ser447_Arg448dup | C | CAGCCGG | INDEL | | inframe_6 | | A | 9537 | 28,47 | 8986 |  |  |  |
| 15 | 90631934 | IDH2 | 4 | c.419G>A | p.Arg140Gln | C | T | SNP | | missense | | A | 8884 | 3,14 | 8986 |  |  |  |
| 18 | 42531917 | SETBP1 | 4 | c.2612T>C | p.Ile871Thr | T | C | SNP | | missense | | A | 7340 | 1,44 | 8986 |  |  |  |
| 20 | 31023453 | ASXL1 | 12 | c.2940dupT | p.Glu981*fs*1 | ATT | ATTT | INDEL | | frameshift | | A | 10554 | 41,54 | E407 |  |  |  |
| 20 | 31024780 | ASXL1 | 12 | c.4265G>C | p.Ser1422Thr | G | C | SNP | | missense | | B | 9017 | 1,05 | E407 |  |  |  |
| 2 | 25457242 | DNMT3A | 23 | c.2645G>A | p.Arg882His | C | T | SNP | | missense | | B | 10354 | 45,65 | E407 |  |  |  |
| 18 | 42531907 | SETBP1 | 4 | c.2602G>A | p.Asp868Asn | G | A | SNP | | missense | | A | 6712 | 44,22 | E407 |  |  |  |
| 4 | 106196680 | TET2 | 11 | c.5015_5018dupTACC | p.Pro1674Thrfs*14 | T | TCTAC | INDEL | | frameshift | | A | 11823 | 37,11 | E409 |  |  |  |
| 2 | 25464445 | DNMT3A | 17 | c.2068G>T | p.Val690Phe | C | A | SNP | | missense | | B | 13967 | 44,52 | E409 |  |  |  |
| 7 | 148516722 | EZH2 | 9 | c.965A>G | p.Asn322Ser | T | C | SNP | | missense | | B | 11547 | 50,92 | E409 |  |  |  |
| 17 | 74732959 | SRSF2 | 1 | c.284C>T | p.Pro95Leu | G | A | SNP | | missense | | B | 9828 | 45,49 | E409 |  |  |  |
| 4 | 106164758 | TET2 | 6 | c.3626T>C | p.Leu1209Pro | T | C | SNP | | missense | | B | 12410 | 45,74 | E409 |  |  |  |
| X | 15841258 | ZRSR2 | 11 | c.1344_1346delCAG | p.Arg449del | CGCA | C | INDEL | | inframe_3 | | B | 5617 | 1,66 | E410 |  |  |  |
| X | 15841230 | ZRSR2 | 11 | c.1338_1343dupGAGCCG | p.Ser447_Arg448dup | C | CAGCCGG | INDEL | | inframe_6 | | A | 5772 | 57,93 | E410 |  |  |  |
| 18 | 42531919 | SETBP1 | 4 | c.2614G>A | p.Gly872Arg | G | A | SNP | | missense | | B | 9263 | 45,35 | E410 |  |  |  |
| 21 | 44514780 | U2AF1 | 6 | c.467G>A | p.Arg156His | C | T | SNP | | missense | | B | 11671 | 39,82 | E410 |  |  |  |
| 21 | 36231859 | RUNX1 | 6 | c.524delT | p.Leu175Argfs*36 | CA | C | INDEL | | frameshift | | A | 11142 | 44,08 | E411 |  |  |  |
| 12 | 25398284 | KRAS | 2 | c.35G>A | p.Gly12Asp | C | T | SNP | | missense | | A | 10932 | 4,02 | E411 |  |  |  |
| 1 | 115256528 | NRAS | 3 | c.183A>T | p.Gln61His | T | A | SNP | | missense | | B | 9691 | 5,6 | E411 |  |  |  |
| 1 | 115258747 | NRAS | 2 | c.35G>A | p.Gly12Asp | C | T | SNP | missense | | A | | | | | 11873 | 26,96 | E411 |

**Supplementary table 3.** Primer sequences used for pyrosequencing analysis. Primers used for IDH1 (R132), SF3B1 (K700), SETBP1 (D868, G870), SRSF2 (P95), TP53 (Y220) pyrosequencing have been previously reported [1].

| **Gene** | **Sequence** | |
| --- | --- | --- |
| **IDH2 R172K** | Fw | 5’- ACATCCCACGCCTAGTCCC -3’ |
|  | Rv | 5’- bio-TCTCCACCCTGGCCTACCTG -3’ |
|  | Seq | 5’- CCCATCACCATTGGC -3’ |
| **TP53 I162N/Y163H** | Fw | 5’- BIO-ACCTGCCCTGTGCAGCTGT -3’ |
|  | Rv | 5’- CAACCTCCGTCATGTGCTGTG -3’ |
|  | Seq | 5’- CCGTCATGTGCTGTGACTG -3’ |
| **TP53 R273H/C277Y** | Fw | 5’- TACTGGGACGGAACAGCTTTGAG -3’ |
|  | Rv | 5’- TGAGGCTCCCCTTTCTTGC-BIO -3’ |
|  | Seq | 5’- GGAACAGCTTTGAGGT-3’ |
| **TP53 P177S** | Fw | 5’- BIO-GCACATGACGGAGGTTGTGA -3’ |
|  | Rv | 5’- GGGCCAGACCATCGCTATC -3’ |
|  | Seq | 5’- AGCAGCGCTCATGGT -3’ |
| **TP53 R306*** | Fw | 5’- ATCTCCGCAAGAAAGGGG -3’ |
|  | Rv | 5’- GGTCTCCTCCACCGCTTCT-BIO -3’ |
|  | Seq | 5’- CCCAGGGAGCACTAA -3’ |
| **TP53 S127F** | Fw | 5’- TGCCGTCTTCCAGTTGCTTTA -3’ |
|  | Rv | 5’- TGCACAGGGCAGGTCTTG-BIO-3’ |
|  | Seq | 5’- GTCTGTGACTTGCACG -3’ |
| **TP53 splice site c.376-1G>A** | Fw | 5’- TGCCGTCTTCCAGTTGCTTTA -3’ |
|  | Rv | 5’- TGCACAGGGCAGGTCTTG-BIO-3’ |
|  | Seq | 5’- CTCTGTCTCCTTCCTCTT -3’ |
| **TP53 S241Y, C242Y** | Fw | 5’- BIO-TGACTGTACCACCATCCACTACAA -3’ |
|  | Rv | 5’- TGGCAAGTGGCTCCTGAC -3’ |
|  | Seq | 5’- CCGGTTCATGCCGCCC -3’ |

**References:**

Fabiani E, Falconi G, Fianchi L, Criscuolo M, Ottone T, Cicconi L *et al.* Clonal evolution in therapy-related neoplasms. *Oncotarget* 2017;**8**:12031-12040.
