# Supplementary material for: Somatic mutations as markers of outcome after azacitidine and allogeneic stem cell transplantation in higher-risk myelodysplastic syndromes
Source: Leukemia. 2018 Oct 5;33(3):785–90. doi: 10.1038/s41375-018-0284-9 (PMC6462855; doi:10.1038/s41375-018-0284-9)
Supplement: Supplementary file 3 — Supplementary figures [file 41375_2018_284_MOESM3_ESM.pptx]

## Slide 1
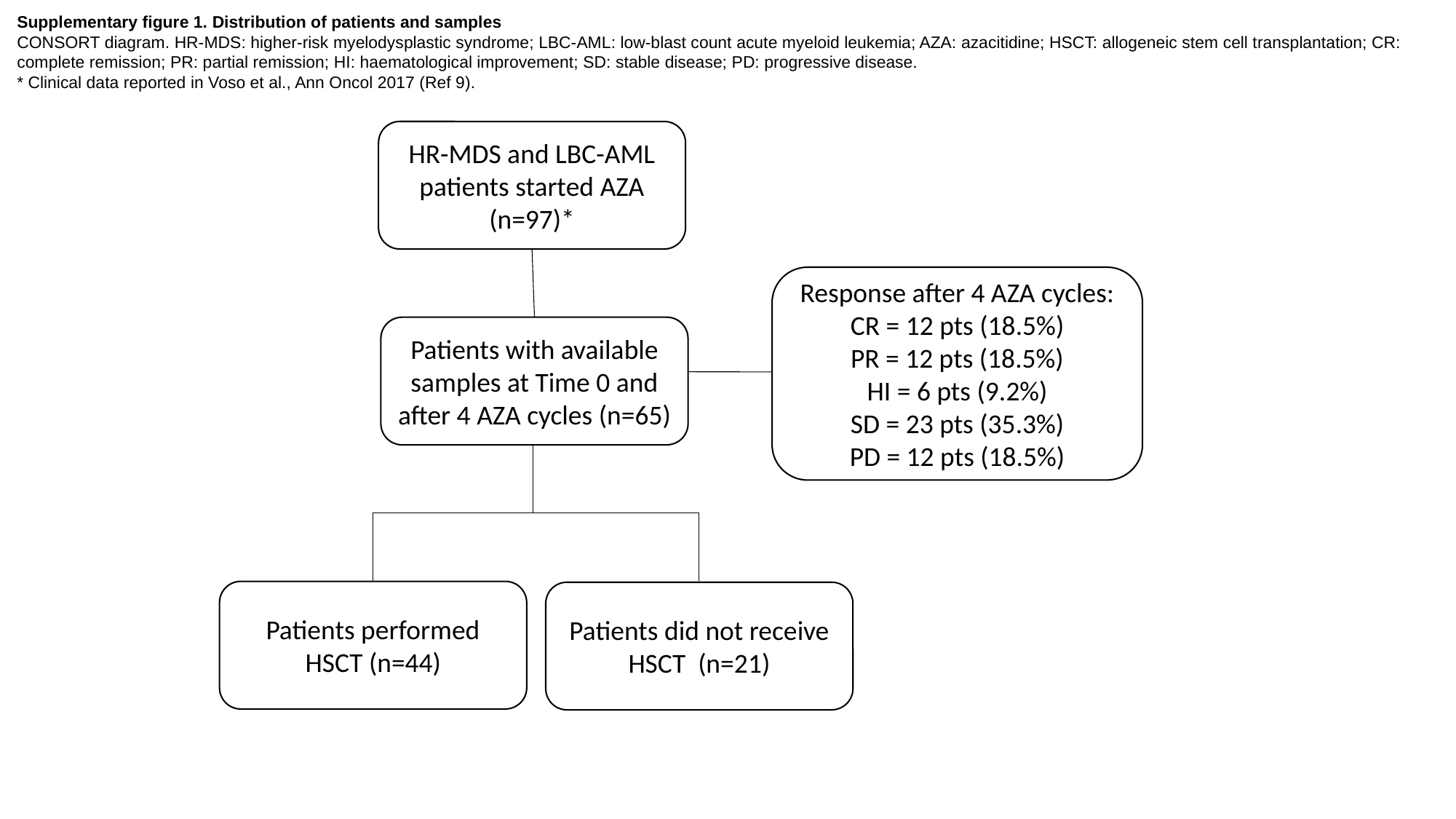

Supplementary figure 1. Distribution of patients and samples
CONSORT diagram. HR-MDS: higher-risk myelodysplastic syndrome; LBC-AML: low-blast count acute myeloid leukemia; AZA: azacitidine; HSCT: allogeneic stem cell transplantation; CR: complete remission; PR: partial remission; HI: haematological improvement; SD: stable disease; PD: progressive disease.
* Clinical data reported in Voso et al., Ann Oncol 2017 (Ref 9).
HR-MDS and LBC-AML patients started AZA (n=97)*
Response after 4 AZA cycles:
CR = 12 pts (18.5%)
PR = 12 pts (18.5%)
HI = 6 pts (9.2%)
SD = 23 pts (35.3%)
PD = 12 pts (18.5%)
Patients with available samples at Time 0 and after 4 AZA cycles (n=65)
Patients performed HSCT (n=44)
Patients did not receive HSCT (n=21)

## Slide 2
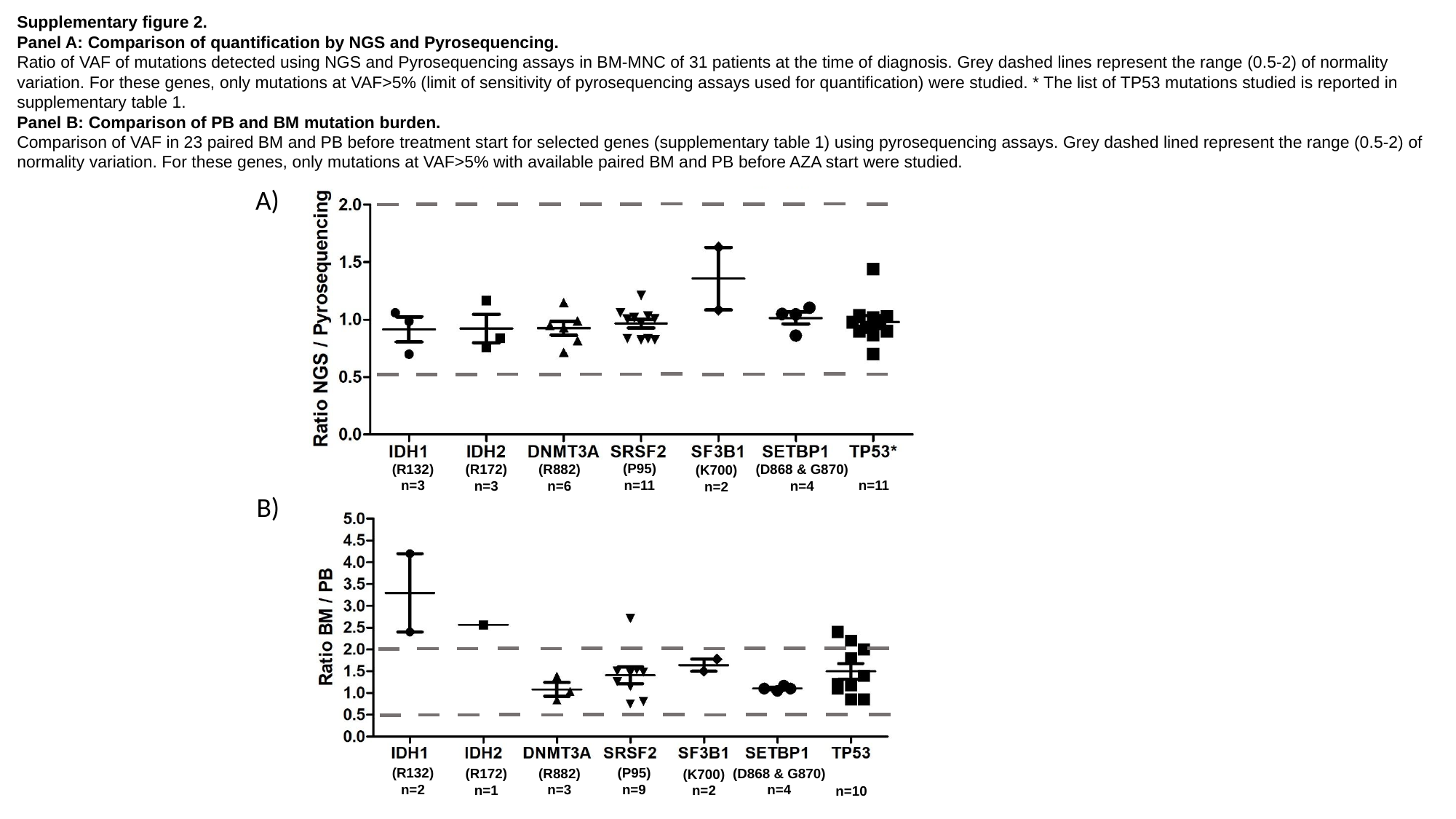

Supplementary figure 2.
Panel A: Comparison of quantification by NGS and Pyrosequencing.
Ratio of VAF of mutations detected using NGS and Pyrosequencing assays in BM-MNC of 31 patients at the time of diagnosis. Grey dashed lines represent the range (0.5-2) of normality variation. For these genes, only mutations at VAF>5% (limit of sensitivity of pyrosequencing assays used for quantification) were studied. * The list of TP53 mutations studied is reported in supplementary table 1.
Panel B: Comparison of PB and BM mutation burden.
Comparison of VAF in 23 paired BM and PB before treatment start for selected genes (supplementary table 1) using pyrosequencing assays. Grey dashed lined represent the range (0.5-2) of normality variation. For these genes, only mutations at VAF>5% with available paired BM and PB before AZA start were studied.
A)
(P95)
n=11
(R132)
n=3
n=11
(D868 & G870)
n=4
(R882)
n=6
(R172)
n=3
(K700)
n=2
B)
(P95)
n=9
(R132)
n=2
(D868 & G870)
n=4
(R882)
n=3
(R172)
n=1
(K700)
n=2
n=10

## Slide 3
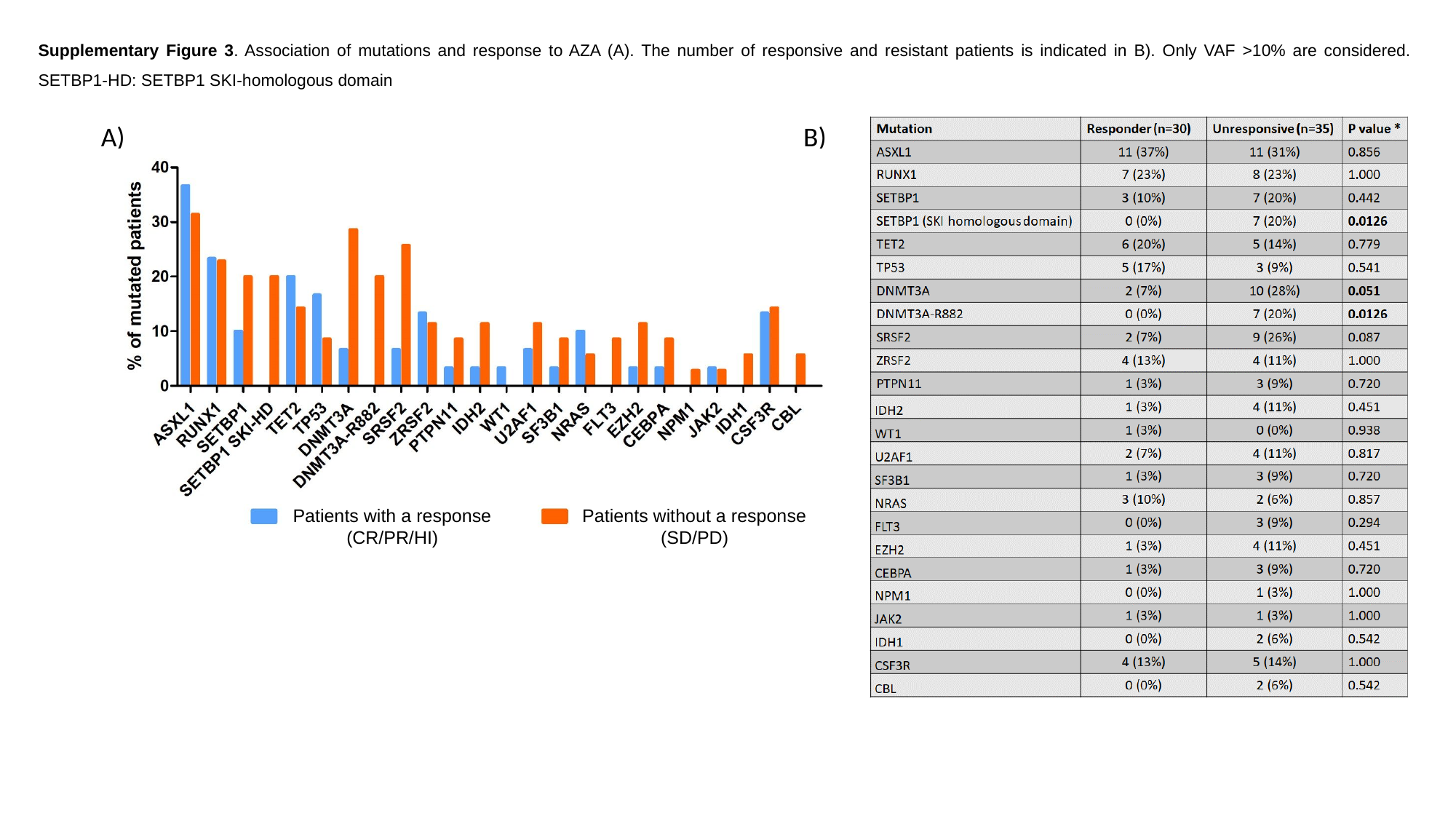

Supplementary Figure 3. Association of mutations and response to AZA (A). The number of responsive and resistant patients is indicated in B). Only VAF >10% are considered. SETBP1-HD: SETBP1 SKI-homologous domain
A)
B)
| |
| --- |
| |
Patients without a response
(SD/PD)
Patients with a response
(CR/PR/HI)

## Slide 4
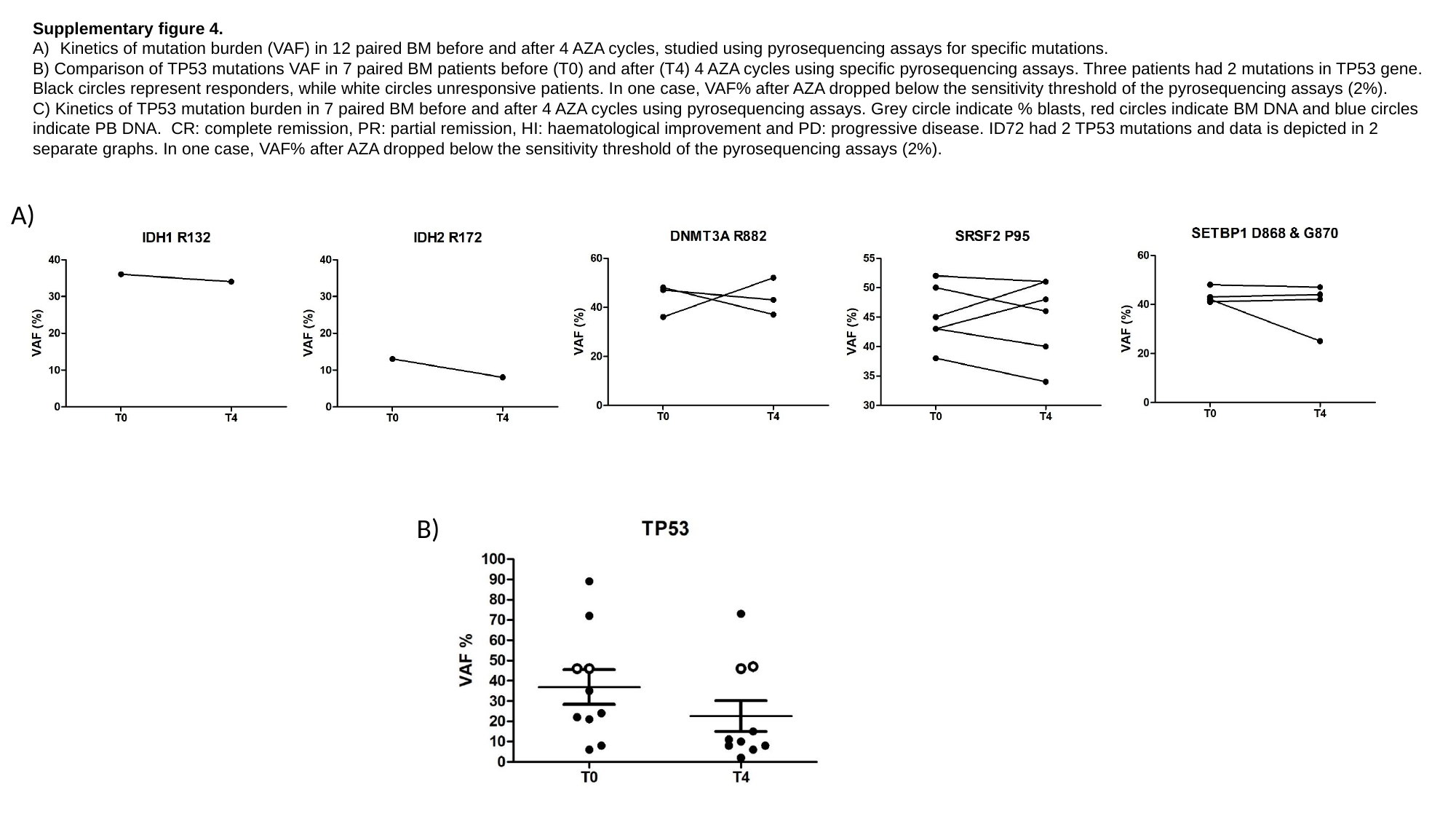

Supplementary figure 4.
Kinetics of mutation burden (VAF) in 12 paired BM before and after 4 AZA cycles, studied using pyrosequencing assays for specific mutations.
B) Comparison of TP53 mutations VAF in 7 paired BM patients before (T0) and after (T4) 4 AZA cycles using specific pyrosequencing assays. Three patients had 2 mutations in TP53 gene. Black circles represent responders, while white circles unresponsive patients. In one case, VAF% after AZA dropped below the sensitivity threshold of the pyrosequencing assays (2%).
C) Kinetics of TP53 mutation burden in 7 paired BM before and after 4 AZA cycles using pyrosequencing assays. Grey circle indicate % blasts, red circles indicate BM DNA and blue circles indicate PB DNA. CR: complete remission, PR: partial remission, HI: haematological improvement and PD: progressive disease. ID72 had 2 TP53 mutations and data is depicted in 2 separate graphs. In one case, VAF% after AZA dropped below the sensitivity threshold of the pyrosequencing assays (2%).
A)
B)

## Slide 5
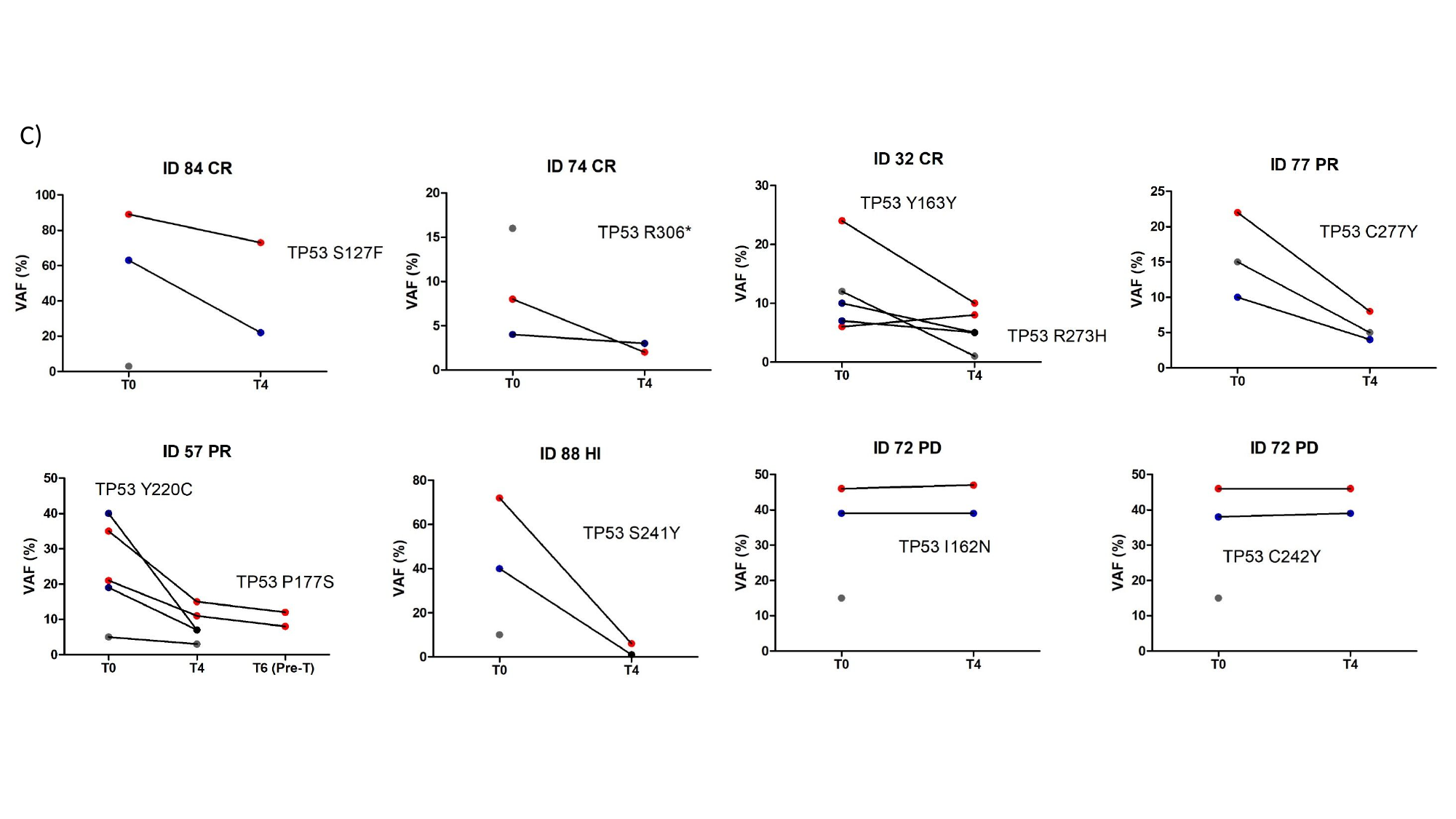

C)

## Slide 6
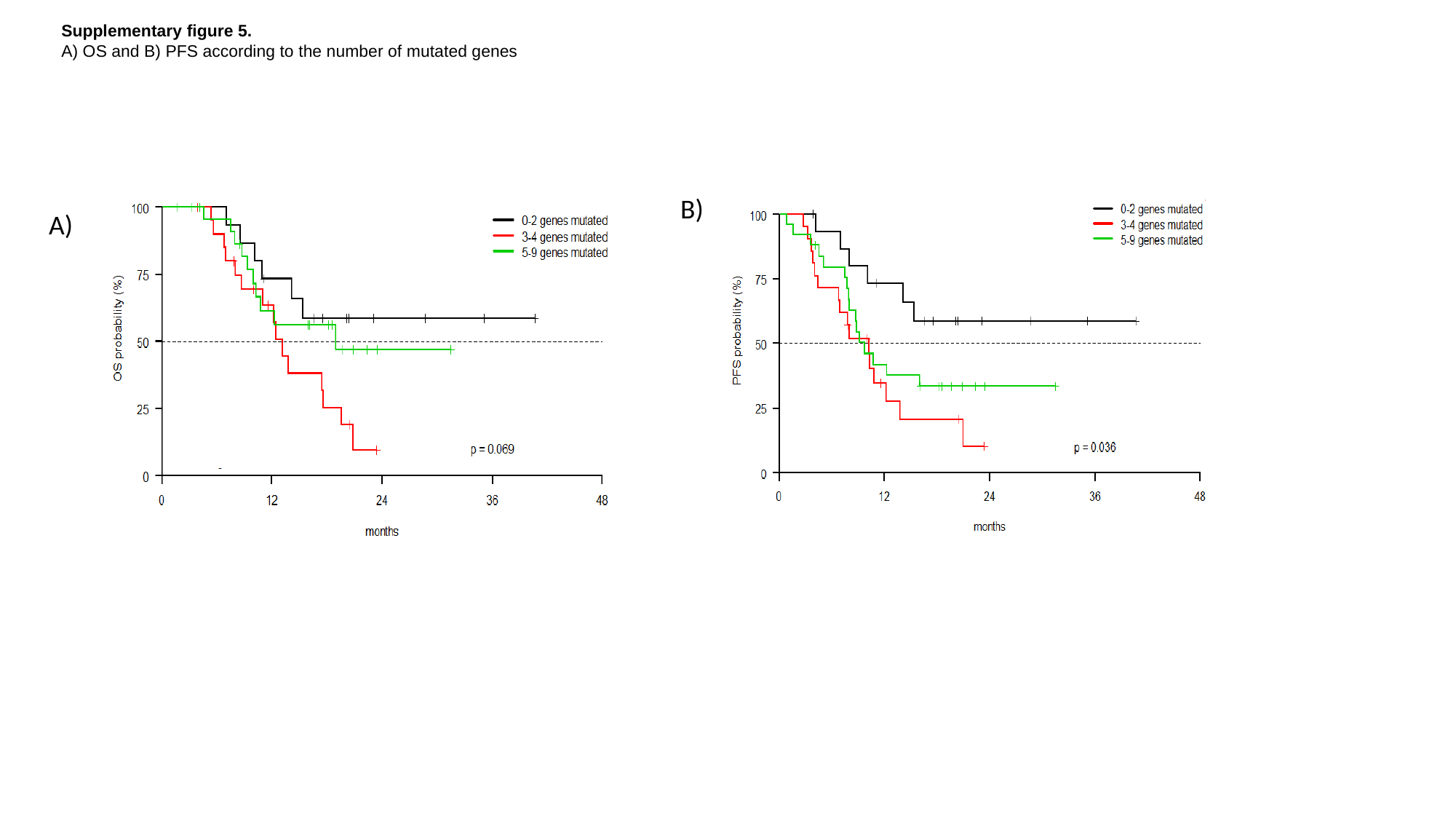

Supplementary figure 5.
A) OS and B) PFS according to the number of mutated genes
B)
A)

## Slide 7
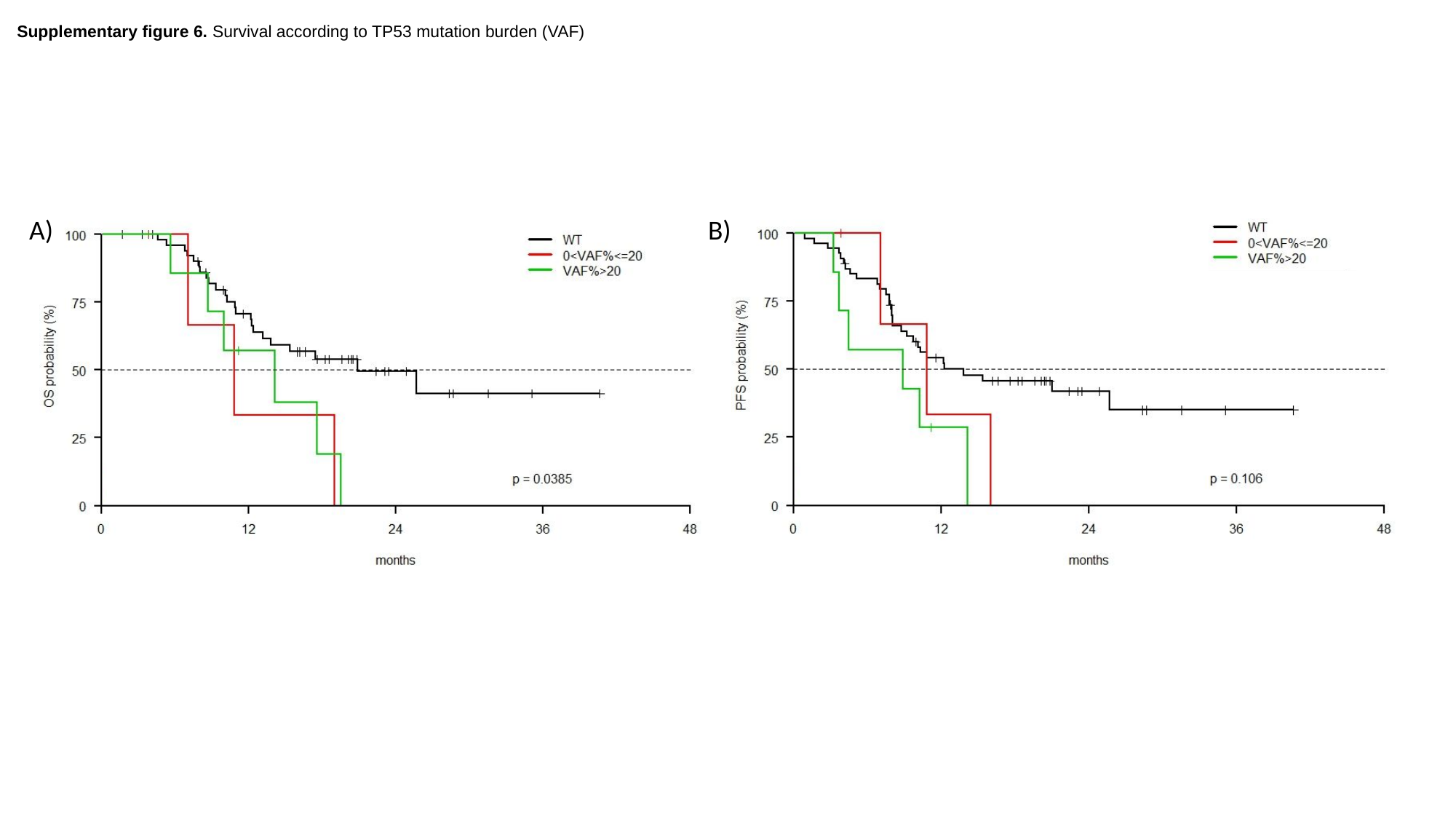

Supplementary figure 6. Survival according to TP53 mutation burden (VAF)
A)
B)
